# From Synthesis to Functionality: Tailored Ionic Liquid-Based Electrospun Fibers with Superior Antimicrobial Properties

**DOI:** 10.3390/polym16152094

**Published:** 2024-07-23

**Authors:** Sanja Rackov, Branka Pilić, Nenad Janković, Marijana Kosanić, Marijana Petković, Milan Vraneš

**Affiliations:** 1 Faculty of Technology Novi Sad, Department of Materials Engineering, University of Novi Sad, Bulevar cara Lazara 1, 21000 Novi Sad, Serbia; brapi@uns.ac.rs; 2 Institute for Information Technologies Kragujevac, University of Kragujevac, Radoja Domanovića 12, 34000 Kragujevac, Serbia; nenad.jankovic@uni.kg.ac.rs; 3 Faculty of Science, Department of Biology and Ecology, University of Kragujevac, Radoja Domanovića 12, 34000 Kragujevac, Serbia; marijana.kosanic@pmf.kg.ac.rs; 4 Department of Atomic Physics, “Vinča” Institute of Nuclear Sciences, National Institute of the Republic of Serbia, University of Belgrade, 11001 Belgrade, Serbia; marijanapetkovic@vin.bg.ac.rs; 5 Faculty of Sciences, Department of Chemistry, Biochemistry and Environmental Protection, University of Novi Sad, Trg Dositeja Obradovića 3, 21000 Novi Sad, Serbia; milan.vranes@dh.uns.ac.rs

**Keywords:** polymeric ionic liquids, electrospinning, nanomaterials, antimicrobial activity

## Abstract

Herein, we report an efficient and facile strategy for the preparation of imidazolium-based ionic liquid (IL) monomers ([C*_n_*VIm][Br], *n* = 2, 4, 6, 8, 10, and 12) and their corresponding polymeric ionic liquids (PILs) with potent antimicrobial activities against Gram-negative and Gram-positive bacteria and fungi. The electrospinning technique was utilized to tailor the polymers with the highest antimicrobial potency into porous membranes that can be easily implemented into diverse systems and extend their practical bactericidal application. The antimicrobial mechanism of obtained ILs, polymers, and nanomaterials is considered concerning the bearing chain length, polymerization process, and applied processing technique that provides a unique fibrous structure. The structure composition was selected due to the well-established inherent amphiphilicity that 1-alkylimidazolium ILs possess, coupled with proven antimicrobial, antiseptic, and antifungal behavior. The customizable nature of ILs and PILs complemented with electrospinning is exploited for the development of innovative antimicrobial performances born from the intrinsic polymer itself, offering solutions to the increasing challenge of bacterial resistance. This study opens up new prospects toward designer membranes providing a complete route in their designing and revolutionizing the approach of fabricating multi-functional systems with tunable physicochemical, surface properties, and interesting morphology.

## 1. Introduction

Infectious diseases continue to be a major public health issue and the leading cause of death. Unfortunately, the unreasonable and excessive use of antibiotics in modern medicine has contributed to the evolution of multi-drug-resistant pathogens that pose another global challenge. Antimicrobial resistance (AMR) refers to the ability of microorganisms, such as bacteria, viruses, fungi, and parasites, to resist the effects of pharmaceuticals designed to kill or inhibit their growth. This resistance makes standard treatments ineffective, leading to the persistence and spread of infections, usually impossible to cure. According to the World Health Organization, AMR is a major challenge facing humanity, with significant negative global consequences [1,2]. AMR is responsible for a large number of deaths, particularly among children, accounting for every fifth death worldwide. Bacterial AMR is the cause of more than 4.95 million deaths, which is significantly higher than the combined number of deaths due to HIV, tuberculosis, and malaria [3]. Poor emission regulations have led to the accumulation of numerous pathogens in the environment [4,5]. For instance, erythromycin, a medication used to fight against infections caused by *Staphylococcus aureus* bacteria, which had already developed resistance to penicillin, quickly lost its effectiveness against 70% of *S. aureus* strains within six months of its initial use in 1952 [6]. Although the WHO has published a high-priority list of critical resistant bacteria (such as *S. aureus*, *E. coli*, etc.) for research and development of new antibiotics [7], it is still quite problematic to address the economics of antimicrobial drug development—the cost of use versus profit [8,9,10]. Since 2010, the UN and WHO have declared Antimicrobial Resistance (ARM) as a fundamental threat [10]. The last report from the EU (Surveillance of Antimicrobial Resistance in Europe, 2023) shows high percentages of AMR to last-line antibiotics (carbapenems) [11]. Considering all the facts mentioned above, there is a significant burden on the need for increased research and development efforts to discover novel antibiotics and other antimicrobial agents. Furthermore, researchers have widely investigated numerous antimicrobial materials such as antimicrobial peptides (AMPs), metal nanoparticles (NPs), cationic polymers, and polymeric nanomaterial strategies to mitigate the spread of antibiotic resistance [12,13,14,15,16].

Ionic liquids (ILs) and polymeric ionic liquids (PILs) are materials that have caught the attention of researchers for developing innovative antimicrobial approaches [17,18,19,20,21,22,23,24]. These liquids can be easily tailored to achieve desired physicochemical and biological properties for specific applications [25,26,27]. This tunable characteristic of ILs and PILs can be exploited to offer innovative antimicrobial strategies to combat the increasing challenge of bacterial resistance. They are considered green and find use in various fields, such as the chemical industry, biomedical applications, air filtration, water treatment, and food packaging [28,29,30]. Although broad spectra of activity against Gram-positive and Gram-negative bacteria and fungi have been known for decades, the exact mechanism of antimicrobial activity of ILs has not been completely elucidated. The main underlying mechanism of ILs antimicrobial efficacy is attributed to their easy attachment to the cell membranes, affecting the diffusion rate. Furthermore, increasing the alkyl chain length increases the lipophilicity and plays a major role in destabilizing and disintegrating the phospholipid bilayer in biological membranes. This behavior is related to the surfactant properties of long alkyl chain ionic liquids that contain *N*-substituted 1-methylimidazolium. These ILs, like [C*_n_*C_1_Im]^+^X^−^ (where *n* > 6), have a charged hydrophilic head group and a hydrophobic tail. They tend to self-assemble and form aggregates, which makes them amphiphilic in nature and exhibit aggregation behavior similar to conventional surfactants. This makes them ideal for designing novel surface-active agents. Some of the imidazolium ILs have been found to have significant biological activity against bacteria due to their amphiphilic nature [31,32,33]. Additionally, imidazolium-based ILs can effectively control the growth of pathogenic, non-pathogenic, and drug-resistant bacteria and fungi [33,34,35,36,37,38]. Studies indicate that imidazolium-based ILs are more effective as antimicrobial agents in biofilm prevention than pyridinium, piperidinium, and pyrrolidinium ILs [34,35]. Biofilm formation and antimicrobial resistance are the primary reasons for persistent tissue infections, medical implants, and wounds [39]. In industrial settings, biofilm formation can result in material corrosion, decreased heat transfer, reduced efficiency of processing equipment, and contamination of the final product [40]. Ionic liquids are an attractive and practical solution for controlling biofilm formation and antibacterial resistance because they may not allow the development of resistance mechanisms. Furthermore, unlike other biocides, they are thermally stable, even at high operating temperatures.

Using ionic liquids in multi-phase processes remains challenging due to their high viscosity, poor mechanical stability, and lack of spatial control, leading to leakage and affecting proper separation from the system. However, these challenges can be overcome by integrating the unique features of ILs into polymers (PILs) via two main synthetic approaches—direct polymerization of IL monomers and chemical modification of polymeric precursors through quaternization reactions. The easy architectural arrangement of the structure of imidazolium-based ILs makes them highly adaptable for various applications beyond their conventional use. PILs combine the unique properties of ionic liquids, such as structure tunability, high ionic conductivity, and thermal stability, along with the processability and mechanical strength of polymers desirable for practical use [41].

There have been many publications regarding the development of innovative imidazolium-based ionic liquids [42,43,44]. However, these compounds continue to inspire scientists toward new synthesis strategies in obtaining corresponding polymeric ionic liquids to meet specific application demands [45]. Smith et al. describe the preparation of thiomidazolium-based PILs effective against *Pseudomonas aeruginosa* with a slow degradation trend in water [46]. Zhou et al. reported the design of semi-interpenetrate network hydrogels including PILs, poly(1-ethyl-3-vinylimidazolium furan-2-carboxylate), and poly(1-butyl-3-vinylimidazolium furan-2-carboxylate) with excellent antibacterial activity against *Escherichia coli* and *Staphylococcus aureus* and anti-inflammatory properties intended for wound healing promotion [47]. The structure–antibacterial activity relationship of imidazolium-based ILs, PILs, and PIL membranes obtained by photo-crosslinking with acrylonitrile and styrene was investigated by Zheng et al. [48]. Hollow polymeric nanosphere-supported imidazolium-based PILs were developed by Zhang et al., and the influence of the alkyl chain length on efficacy against *Escherichia coli* and *Bacillus subtilis* was investigated [18]. Choosing an appropriate processing technique is essential in creating a material with a well-defined structure. Besides being tunable in nature, the form of the resulting PIL should also be well-defined. In this sense, electrospinning as a versatile and sophisticated technique allows scalable and low-cost production of fibrous materials with diameters ranging from the micro to the nanoscale. Applying high voltage to a polymer solution provides a unique architecture with tunable morphology (porosity, fiber diameter) that is impossible with other processing techniques [49]. These continuous, interconnected ultrathin materials have a strong tendency to adhere due to their high surface-to-volume ratios, which provide a greater functional contact area with targeted microbes and reach the long-term effect of the investigated polymer. However, few examples in the literature deal with PIL in the form of fibrous membranes [50,51,52]. As a result, the electrospinning technique provides an entirely new approach to developing PIL-based membrane systems and expanding future perspectives.

This study focuses on the synthetic process of a range of monomer ionic liquids named 1-alkyl-3-vinylimidazolium bromide ([C*_n_*VIm][Br], *n* = 2, 4, 6, 8, 10, and 12) and their corresponding polymeric counterparts, Poly([C*_n_*VImBr]), through a radical polymerization process. The aim is to further develop PILs as multifunctional materials at the micro and nanoscale through electrospinning. The main advantage of the obtained electrospun materials over others of its kind is that the polymer matrix does not require the incorporation of active components or to be loaded by antimicrobial agents to exhibit exceptional capability against a wide range of microorganisms. Thus, representing a cost-effective one-component system avoiding leaking, migration, or evaporation of the active compound.

The specific objective is to compare and evaluate the antimicrobial potency of these materials using standardized procedures and to gain a deeper understanding of the differences in antimicrobial activity between imidazolium-based ILs, their corresponding polymers, and the resulting electrospun materials. Additionally, the impact of the alkyl chain length on their activity needs to be determined.

The presented study is a significant breakthrough in the PILs research field. It focuses on demonstrating the electrospinning of these liquids to create a novel material with potent antimicrobial properties. This research paves the way for exploring new applications in science and technology. The results of this study showcase the potential of PILs as potent molecules against microorganisms and an important first step toward unlocking the full potential of this innovative material. 

## 2. Materials and Methods

### 2.1. Materials

1-Vinylimidazole (≥99%), bromoethane (98%), 1-bromobutane (≥99%), 1-brohohexane (≥98%), 1-bromooctane (99%), 1-bromodecane (98%), 1-bromododecane (98%), ethyl-acetate (≥99.8%), chloroform (99.8%), N,N-dimethylformamide (DMF) (HPLC grade, ≥99.9%), methanol (≥99.8%), dimethyl sulfoxide (≥99.7%), and azobisisobutyronitrile (AIBN) (97%) were purchased from Sigma-Aldrich (St. Louis, MO, USA) and used as received without further purification.

### 2.2. General Synthetic Procedure for the 1-Alkyl-3-vinylimidazolium Bromide, Poly(1-alkyl-3-vinylimidazolium bromide), and Preparation of Solutions for Electrospinning

The synthetic method of 1-alkyl-3-vinylimidazolium bromide ([C*_n_*VIm][Br], *n* = 2, 4, 6, 8, 10, and 12) included the addition of 1-vinylimidazole and 1-alkylbromides in a round-bottom flask fitted with a reflux condenser at a molar ratio of 1:1.2 (according to previously established procedures [53]). The mixture was dissolved in ethyl acetate and stirred at 110 °C for 24–48 h. The top layer of the solvent containing the unreacted chemicals was removed, and the resulting IL was purified three times with ethyl-acetate. The residual organic solvent was then removed using rotary evaporation and stored over P_2_O_5_ in a vacuum desiccator. The structure (Table 1) confirmation of the synthesized IL was carried out by ^1^H NMR and FTIR analysis (Figures S1–S12 in the Supplementary Materials).

The homopolymer PILs, Poly([C*_n_*VImBr], *n* = 2, 4, 6, 8, 10, and 12), were synthesized by free radical polymerization (the structures are given in Table 1). The polymerization process was carried out in a round-bottom flask equipped with a reflux condenser with gas inlet valve. The reaction mixture containing a proper amount of IL monomer and chloroform was purged with N_2_ for 20 min. Radical initiator AIBN (1 wt.% with respect to IL) was added and immersed in an oil bath at 60 °C for 6 h. The obtained polymer was diluted and purified with chloroform three times and dried under vacuum at 60 °C. FTIR spectroscopy and MS have carried out the structure confirmation (Figures S13–S18 and S21–S28 together with their assignments in the Supplementary Materials).

In the next phase, the initial solution for electrospinning of pure Poly([C_10_VIm][Br]) is prepared by dissolving in DMF (and several drops of methanol) at a weight concentration of 30 wt.%. A 15 wt.% solution of Poly([C_12_VIm][Br]) is obtained by dissolution in the mixture of chloroform/DMF (8:2). The solutions were stirred over 24 h before the electrospinning process.

### 2.3. Microbial Strains and Culture Conditions

Antimicrobial activities of tested compounds were evaluated against the following five strains of bacteria: *Staphylococcus aureus* (ATCC 25923), *Bacillus subtilis* (ATCC 6633), *Klebsiella pneumoniae* (ATCC 13883), *Escherichia coli* (ATCC 25922), and *Proteus mirabilis* (ATCC 29906) and five species of fungi: *Aspergillus niger* (ATCC 16888), *Aspergillus flavus* (ATCC 9170), *Penicillium italicum* (ATCC 10454), *Mucor mucedo* (ATCC 20094), and *Trichophyton mentagrophytes* (ATCC 9533), obtained from the American Type Culture Collection (ATCC) (Manassas, VA, USA). 

The bacteria isolates were picked from overnight cultures in Mueller–Hinton agar, and suspensions were prepared in sterile distilled water. The turbidity of suspensions was adjusted by comparing with 0.5 McFarland’s standard to approximately 10^8^ CFU/mL. Fungal suspensions were prepared from 5- to 8-day-old cultures that grew on a Sabouraud dextrose agar. Fungal suspensions consisted of fungal spores and sterile distilled water, adjusted to contain approximately 10^6^ CFU/mL.

### 2.4. Minimal Inhibitory Concentration (MIC) Determination

The 96-well microtiter assay using resazurin as the indicator of cell growth [54] was employed to determine the minimum inhibitory concentration (MIC) of the active components. Starting solutions of tested compounds were obtained by dissolving them in 5% DMSO. Next, serial twofold dilutions of tested compounds were made in sterile 96-well plates containing Mueller–Hinton broth for bacterial cultures and a Sabouraud dextrose broth for fungal cultures. After that, diluted bacterial and fungal suspensions were added to appropriate wells, and finally, resazurin solution, as an indicator of bacterial growth, was added to each well inoculated with bacteria. The inoculated plates were incubated at 37 °C for 24 h for bacteria and 28 °C for 72 h for fungi. The MIC was determined visually and defined as the lowest concentration of tested compounds that prevented resazurin color change from blue to pink. For fungi, MIC values of the tested substance were determined as the lowest concentration that visibly inhibited mycelia growth. Streptomycin, Doxycycline (for bacteria), Ketoconazole, and Fluconazole (for fungi) were used as positive controls. A solvent control test was performed to study the effect of 5% DMSO on the growth of microorganisms.

### 2.5. Apparatus and Procedures

Electrospinning equipment. The electrospinning process was performed using Fluidnatek LE-10 (setup by BioInicia, Valencia, Spain) after optimization under defined constant ambient conditions (relative humidity 40 ± 5% and temperature 25 ± 2 °C). The two solutions were loaded into a 5 mL plastic syringe fitted with a blunt stainless steel needle serving as the nozzle and connected to the high-voltage supply. The grounded collecting plate was covered with aluminum foil. The solution flow rate was adjusted with a syringe pump in the 0.3–0.5 mL/h range under the applied electrical potential difference of 20–27 kV and nozzle to collector distance of 10 cm. For the solution of Poly([C_10_VIm][Br]), a positive voltage ranging from 20–22 kV was applied, while for Poly([C_12_VIm][Br]), it was higher (27 kV).

Nuclear Magnetic Resonance (NMR) spectra. ^1^H NMR spectroscopy was performed by applying a Bruker Avance III 400 MHz spectrometer (Billerica, MA, USA) using D_2_O or CDCl_3_ at *T* = 25 °C. The tetramethylsilane was used as an accepted internal standard for calibrating chemical shifts for ^1^H. ^1^H homodecoupling and 2D COSY methods were used routinely to assign obtained NMR spectra.

Matrix-assisted laser desorption and ionization Time-of-Flight mass spectrometry (MALDI TOF MS). The molecular weight analysis of the synthesized polymers was performed on an Axima Performance device from Shimadzu (Shimadzu Europe, Duisburg, Germany). The instrument was equipped with a variable repetition rate nitrogen laser with a maximum frequency of 50 Hz. The spectra (Figures S21–S28 in the Supplementary Materials) were acquired either in the positive and reflectron mode with the *m*/*z* ratio from 300 to 1500 with α-hydroxycinnamic acid (CHCA) or in the linear mode, up to 50,000 *m*/*z* ratio (with synapinic acid, SA) as matrix. The matrices were prepared at a concentration of 10 mM in 50% acetonitrile in water, supplemented with 0.01% trifluoroacetic acid (TFA) to increase the ionization. Polymers were dissolved at 1 mg/mL concentration and mixed with the matrix solution at the 1:1 volume ratio. Afterwards, 1 µL of the sample/matrix mixture was applied to the sample plate and left to dry at room temperature. Each spectrum represents an average of a minimum of 100 individual laser shots. Only the signals that differ from the background matrix signals are indicated according to their position.

Vibrational Infrared (IR) spectra. The chemical structure of the samples was examined with Shimadzu IRaffinity-1s Fourier Transform Infrared Spectrometer (Kyoto, Japan) with Attenuated Total Reflectance (MIRacle 10 ATR; Dia/ZnSe) measurement mode in the mid-region (4000 to 400 cm^−1^). The measurements were performed with a total of 60 scans and a spectral resolution of 4 cm^−1^. 

Differential Scanning Calorimetry (DSC). Thermal properties were evaluated using TA Instruments differential scanning calorimeter DSC Q20 (New Castle, DE, USA) at a heating rate of 10 °C·min^−1^ with a 50 cm^3^·min^−1^ nitrogen flow rate. The samples (about 2 mg) were hermetically sealed in aluminum DSC pans, and the corresponding thermograms were obtained during heating from 25 °C to 100 °C followed by a cooling cycle to −90 °C, then reheating up to 300 °C with standard temperature uncertainty *u*(*T*) = ±0.5 °C. Indium reference samples were used for temperature and cell constant calibration, while specific heat capacity (*C*_p_) calibration was performed via sapphire crystal reference, both provided by TA Instruments. DSC thermograms were analyzed using TA Universal Analysis 2000, V4.5A, build 4.5.0.5.

Scanning Electron Microscopy (SEM). The morphology of nanofibers was evaluated using scanning electron microscopy with the help of JEOL JSM-6460 (Tokyo, Japan). Fiber mats of 0.5 cm × 0.5 cm were vacuumed, gold-sputter coated, and exposed to an accelerating voltage of 20 kV. The average diameter of the fibers was calculated using ImageJ software version 1.54 by randomly selecting 100 fiber segments from micrographs.

Water Contact Angle (WCA). Wettability of the electrospun fibers was investigated by the sessile drop method, placing a drop of distilled water onto the surface of the samples. Five measurements were performed for each sample using an Ossila optical goniometer (Sheffield, UK) at room temperature (25 ± 1 °C). Water contact angle was determined as the mean value of the left and right angle between the water drop and the fibers using Ossila Contact Angle software, version 1.3.0.0.

## 3. Results and Discussion

### 3.1. Synthetic Procedure and Structural Characterization of Poly(1-alkyl-3-vinylimidazolium bromide)

It is rare to find examples that describe the effectiveness of micro and nanoscale materials made from ionic liquids against microbial species. This is particularly challenging when creating materials via electrospinning from pure polymeric ionic liquid solutions. The charged nature of PILs consisting of ionic species in each repeating unit can cause repulsion interactions of the charged moieties, making it difficult to produce these materials. Although the properties of these nanomaterials can be adjusted by modifying the structure of the initial ILs, only a few authors [50,51] have reported examples of PIL fibers made through electrospinning without blending with other supporting, more neutral polymers [52,55,56]. Since PILs are generally a new class of polymers, understanding their behavior in solution is not as developed as it is for neutral polymers. This challenge led us to conduct research to obtain a unique architecture based on PILs, expand the utility of ILs beyond their conventional applications, and evaluate the influence of the nanoscale on antimicrobial activity.

**Scheme 1:**
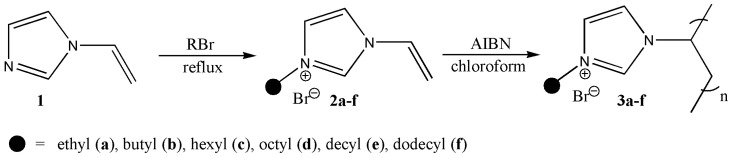
Synthetic line towards PILs of **3a**–**f**.

To address the challenges described above, we have started with direct quaternization of 1-vinyl imidazole (**1**) via *N*-alkylation using alkyl halides (RBr, **a**–**f**, Scheme 1). Almost-quantitative (>99%) yields of **2a**–**f** were achieved in all cases. In the literature, quaternization initiated from poly(vinyl imidazole) (not from monomer 1) to PILs was achieved in the range of 90–98%, implying that our strategy gave a better conversion percentage in this process [48].

**Figure 1:**
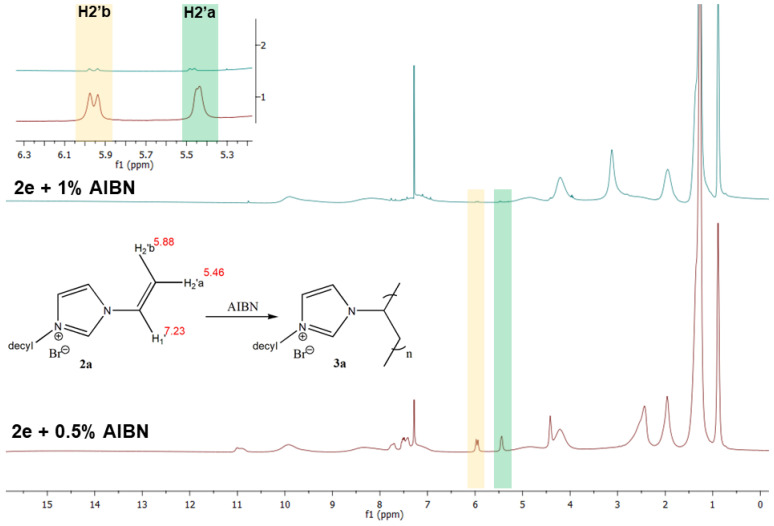
Stacked NMR spectrum for optimization of polymerization of [C_10_VIm][Br] with: 1% AIBN (blue spectrum) and 0.5% AIBN (red spectrum) (**2e**).

Therefore, the optimization of the free radical polymerization process was necessary in the second synthetic step. We use different percentages of the radical initiator AIBN (0.1–1%) for direct polymerization of ILs bearing the decyl chain [C_10_VIm][Br] (**2e**) in chloroform. Attempts with AIBN content below 0.5% were unsatisfactory. For better convenience, two samples of a crude mixture of Poly([C_10_VIm][Br]) (**3e**) after polymerization with 0.5 or 1% of AIBN were depicted in Figure 1. Stacked NMR spectra of **3e** showed significant and different effects of AIBN content on the polymerization of **2e**. Applying 0.5% of AIBN could not finish the polymerization process of **2e**, which was observed in NMR spectra. Based on MS spectra, 0.1% and 0.5% of AIBN gave appropriate dimer and trimer of **2e** (Figures S25 and S26, respectively). However, the absence of protons from vinyl function located at 5.46 (=CH_2_) and 5.88 (N-CH=) ppm implies successful polymerization of **2e** with 1% of AIBN. In addition, in the MS spectrum (Figure S27) of the same sample, we found ion at 15,317 *m*/*z* (n = 48.6). Generally, from MS spectrum, the number of monomeric units and the molecular mass increase in correlation with the number of carbon atoms in the chain. For example, the molecular weights of **3a** (ethyl), **3b** (butyl), and **3c** (hexyl) are 3682.3, 3686.1, and 4327.3, respectively. Octyl (**3d**), decyl (**3e**), and dodecyl (**3f**) have molecular weights that are significantly higher compared to **3a**–**c** (15,310.6, 15,317.4, and 11,188.9, respectively). With these optimized conditions, we checked the substrate scope of all ILs.

### 3.2. Thermal Properties of Poly(1-alkyl-3-vinylimidazolium bromide)

**Figure 2:**
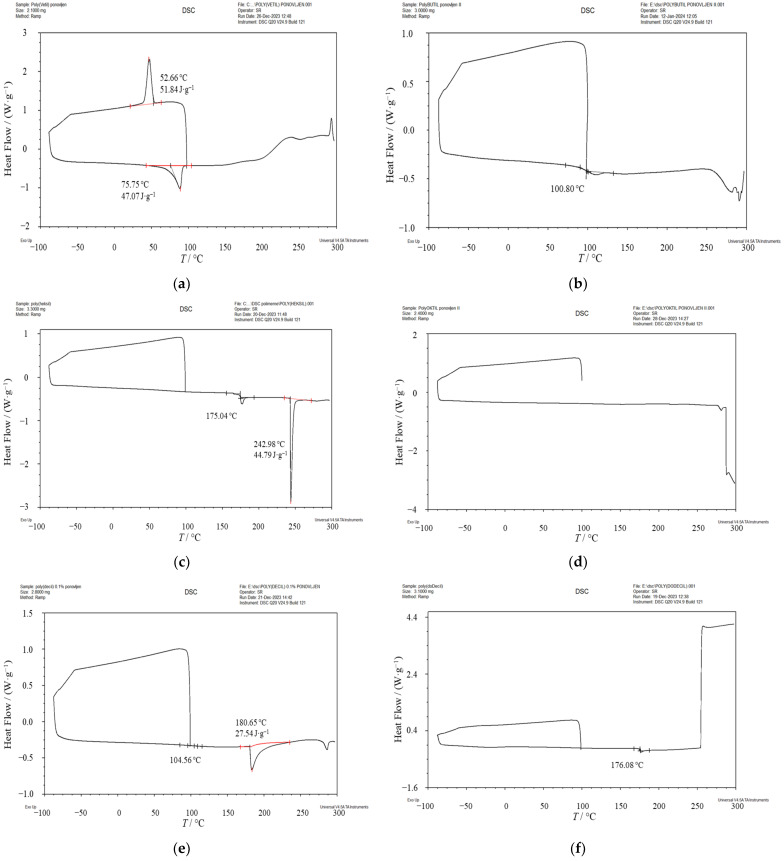
DSC thermograms of cooling and reheating cycles for: (**a**) Poly([C_2_VIm][Br]); (**b**) Poly([C_4_VIm][Br]); (**c**) Poly([C_6_VIm][Br]); (**d**) Poly([C_8_VIm][Br]); (**e**) Poly([C_10_VIm][Br]); and (**f**) Poly([C_12_VIm][Br]).

DSC measurements were performed to investigate the thermal properties and determine the phase change behavior of the prepared materials. It is essential for a deeper understanding of their final features and applicability. Figure 2a–f displays the cooling and reheating step curves for poly(1-alkyl-3-vinylimidazolium bromide). The same conditions are applied to their corresponding monomer ILs, and the results are published within our previous research but are also shown in Table 2 [53]. 

As evidenced in Table 2 and Figure 2, [C_2_VIm][Br] and Poly([C_2_VIm][Br]) show similar thermal behavior with the appearance of crystallization and melting phase changes in the temperature range from 35 °C to 75 °C. While liquid subcooling to an amorphous glass for ILs [C_4_VIm][Br], [C_6_VIm][Br], [C_8_VIm][Br], and [C_10_VIm][Br] appears at relatively low temperatures below −40 °C, glass transitions for Poly([C_4_VIm][Br]), Poly([C_6_VIm][Br]), Poly([C_10_VIm][Br]), and Poly([C_12_VIm][Br]) occur at 100.80 °C, 175.04 °C, 104.56 °C, and 176.08 °C, respectively. These values are in good agreement with the *T*_g_ values reported for vinylimidazolium polymers [57]. Agapov examined the impact of the monomer’s polarity and the position of the polar group on the chain dynamics and the glass transition [58]. Relatively high values for *T*_g_ can be explained by polar interactions influencing the conformational rigidity. The DSC curve for Poly([C_8_VIm][Br]) revealed no phase change at the applied thermal treatment under 260 °C associated with the onset of thermal degradation.

### 3.3. Chemical Structure Evaluation of IL, PIL, and Electrospun Fibers

As previously mentioned, the elongation of the alkyl chain in ionic liquids leads to lower MIC values. This same trend is expected for their polymeric analogs. Therefore, Poly([C_10_VIm][Br]) and Poly([C_12_VIm][Br]) were chosen for the production of fibrous membranes. 

**Figure 3:**
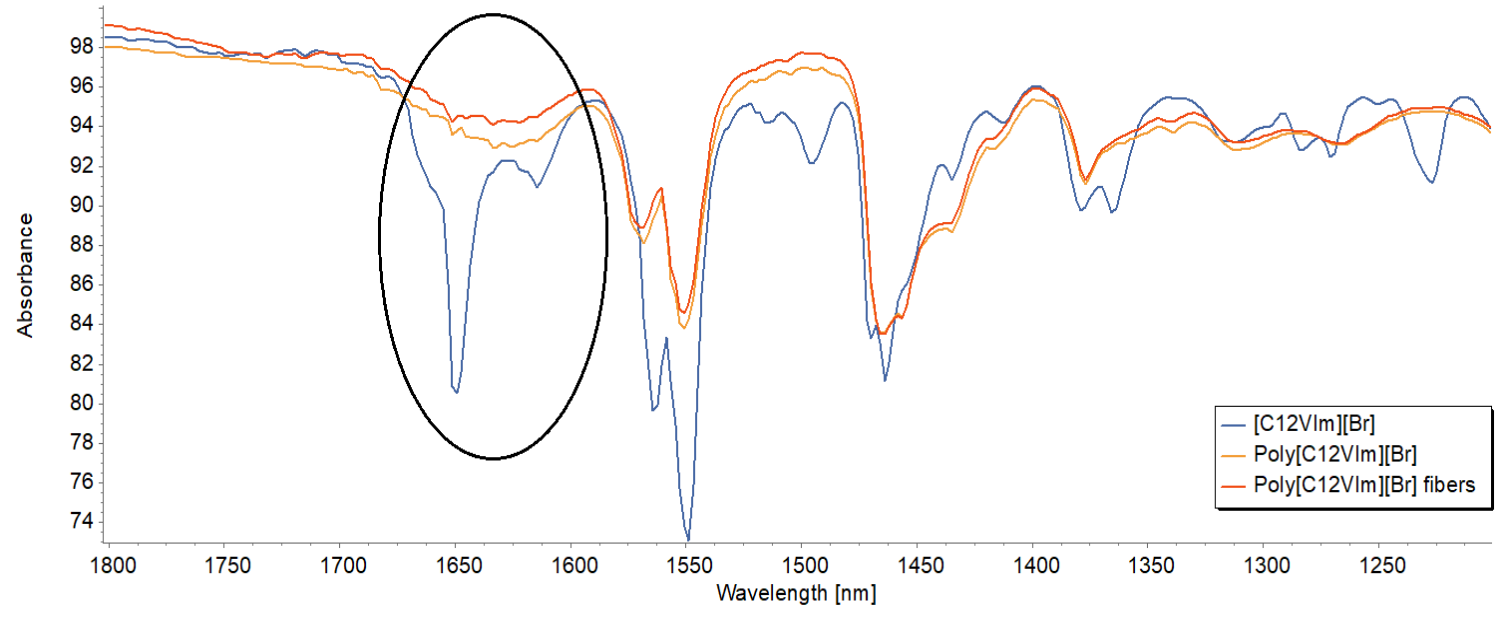
Overlapped IR spectrum of selected monomer [C_12_VIm][Br], polymer Poly[C_12_VIm][Br], and its electrospun fibers.

After determining the efficient electrospinning conditions, the fibrous membranes were prepared following the procedure outlined in Section 2.5. The polymer membranes, consisting of a dense layer of fibers, were electrospun onto an aluminum foil. Their chemical structure was confirmed through FTIR analysis (Figures S19 and S20). To represent the efficacy of the electrospinning process, a comparison between FTIR spectra of IL, PIL, and PIL electrospun fibers was made. Monomer, [C_12_VIm][Br], and its PIL, Poly([C_12_VIm][Br]), showed different IR bands in the double bond area. Double bond stretching vibration of [C_12_VIm][Br] occurs around 1650 cm^−1^. Furthermore, at lower frequencies, around 950 cm^−1^, CH=CH_2_ out-of-plane bending vibrations are found. As expected, those vibrations were missed from the IR spectrum of Poly[C_12_VIm][Br]. Poly[C_12_VIm][Br] and its electrospun fibers have similar IR spectra (Figure 3), implying that no structural changes occurred during the electrospinning process.

### 3.4. Morphology and Wettability Evaluation of the Electrospun Fibers

The morphologies of the fibers were observed through scanning electron microscopy (SEM) micrographs. The wettability of the obtained membranes has also been investigated to provide further insight into the interactions of these materials with water. This analysis offers valuable information about their behavior when in contact with liquids.

**Figure 4:**
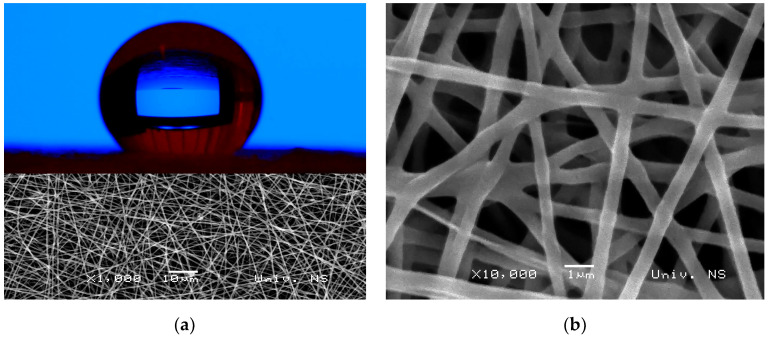
Contact surface of a water droplet and Poly([C_10_VIm][Br]) fibers and SEM micrographs of Poly([C_10_VIm][Br]) fibers at different magnifications: (**a**) ×1000 and (**b**) ×10,000.

**Figure 5:**
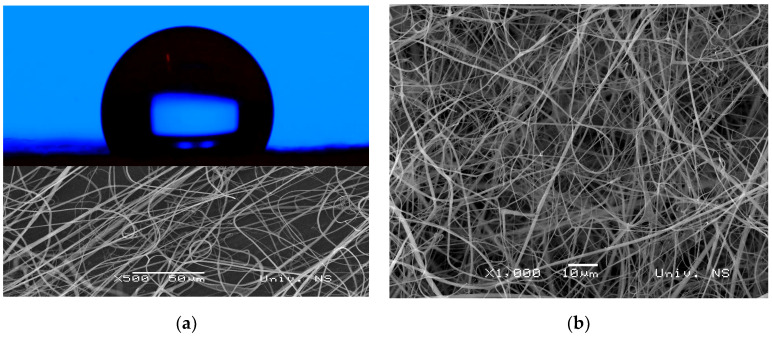
Contact surface of a water droplet and Poly([C_12_VIm][Br]) fibers and SEM micrographs of Poly([C_12_VIm][Br]) fibers at different magnifications: (**a**) ×500 and (**b**) ×1000.

SEM micrographs of the electrospun fibers of Poly([C_10_VIm][Br]) and Poly([C_12_VIm][Br]) are displayed in Figure 4 and Figure 5 and as well in the Supplementary Materials Figures S29 and S30, respectively, at different magnifications. Randomly oriented nanofibers of Poly([C_10_VIm][Br]) depicted the interconnected structure without the occurrence of irregularities and with an average diameter size varying from about 240 to 520 nm. Figure 4a also displays the contact surface between a water droplet and Poly([C_10_VIm][Br]) nanofibers with a contact angle of 132.85°, describing highly hydrophobic materials. This behavior is expected due to long (decyl) alkyl chains within the polymer structure. Hydrophobic nature ensures their application in different antimicrobial systems, which include high humidity conditions, without change in the initial structure of the membranes, dissolution, or leaking from the site of their application. Furthermore, hydrophobic materials prevent microbes from adhesion owing to their water-repellent nature, thereby creating protection from bacterial biofilm formation and preventing them from establishing a foothold on the surface [59]. This may be also advantageous in high-temperature separations or processes where water presence is undesirable.

In the case of Poly([C_12_VIm][Br]), fibers are randomly oriented, continuous, and mostly cylindrical-shaped with the appearance of ribbon-like segments (Figure 5). The fiber diameters and widths of the ribbon-like segments were measured from different sample sections, ranging from approximately 550 to 1650 nm. Other authors also observed similar morphology and diameter size distributions in electrospun cellulose from ionic liquid solvents [60]. Elevated temperatures and amplitude of applied voltage may accelerate the evaporation of solvent molecules and favor the formation of flat-shaped fibers [61]. The high voltage required for fiber production greatly affects the formation of more than one spinning jet around the needle tip during the electrospinning process, weakening the electrical field for each jet. It consequently leads to a wide-diameter distribution [62]. Despite expectations that an increase in the alkyl chain length in Poly([C_12_VIm][Br]) fibers would lead to more hydrophobic behavior compared to Poly([C_10_VIm][Br]), the opposite occurred. A slight enhancement in wettability and a decrease in the water contact angle were observed for Poly([C_12_VIm][Br]) fibers, resulting in a value of 120.72°. This phenomenon can be attributed to differences in surface morphology and interfibrillar spaces, unlike those in Poly([C_10_VIm][Br]). The broad diameter distribution and ribbon-like segments of the fibers are believed to influence the wettability of the electrospun matrices, thereby reducing the overall water contact angle (WCA). However, their wettability slightly changes with the change in the morphology of the fibers. A delicate balance of hydrophilicity/hydrophobicity of the final membranes can be achieved by combining different PILs in the electrospinning solution. This may be advantageous to optimize their performance for specific application requirements.

### 3.5. Antimicrobial Properties of ILs, PILs, and Electrospun Fibers

Antibacterial and antifungal activities of synthesized imidazolium-based ILs, their corresponding polymers, and electrospun membranes were examined using Gram-positive bacteria *Staphylococcus aureus*, *Bacillus subtilis*, and Gram-negative bacteria *Klebsiella pneumoniae*, *Escherichia coli*, *Proteus mirabilis,* along with fungi, *Aspergillus niger*, *Aspergillus flavus*, *Penicillium italicum*, *Mucor mucedo,* and *Trichophyton mentagrophytes* as model microorganisms. The determined average minimal inhibitory concentration (MIC) values were summarized in Table 3 (for comparative purposes, MIC values for Streptomycin and Doxycycline are also listed) and Table 4 (Ketoconazole and Fluconazole are used as controls).

Considering the results in Table 3 and Table 4, the synthesized compounds ILs and PILs have potent antibacterial and antifungal properties. The MIC values of our compound for the tested bacteria ranged from 0.001 to 1.66 mg/mL, with the lowest value (0.001 mg/mL) being observed against *Bacillus subtilis*, indicating the best antibacterial activity of [C_12_VIm][Br]. After treating fungi with PILs, the antifungal activity ranged from 0.01 to 26.46 mg/mL. *Trichophyton mentagrophytes* showed highest sensitivity after treatment with [C_12_VIm][Br] (0.01 mg/mL). This fungus is a common global pathogen that causes severe skin infections and dermatophytosis in humans and animals. Remarkably low MIC values for both antibacterial and antifungal activities were recorded for ([C_12_VIm][Br]) and their corresponding polymer Poly([C_12_VIm][Br]) and Poly([C_12_VIm][Br]) fibers, which were comparable to the most commonly used antibiotic medications.

The antimicrobial properties of ionic liquids are due to their interaction with cell membranes. This interaction occurs through adsorption, electrostatic attraction, and penetration. These processes lead to the deactivation of membrane proteins and the disintegration of the phospholipid bilayer, causing the cells to become unable to function normally [24]. Ionic liquids can also cause cell lysis and leakage of intracellular cytoplasm.

The cell membrane is a potential target of ionic liquids. Ionic liquids can cross bacterial membranes and enter the cytosol, where they can alter the membrane properties of the bacterial cell wall. These properties include membrane potential, fluidity, viscoelasticity, and the arrangement of phospholipids. By changing the fluidity of the cell membrane, the diffusion rate and stability of the proteins within the membrane are affected, which can severely impact membrane function, including molecule transportation, recognition, migration, adhesion, and mechanotransduction. Additionally, ionic liquids can create pores that result in irreversible damage to the cell membrane, altering its permeability [24,48,63]. Some studies have also shown that imidazolium ionic liquids can decrease the content of ergosterol, a crucial component of fungal cell membranes, which is essential for maintaining membrane integrity and other functions [63,64]. The antifungal activity of imidazolium ionic liquid compounds has been demonstrated by affecting various cellular processes such as cell volume reduction, intracellular ROS production, and mitochondrial dysfunction [63]. Moreover, inhibition of conidia germination and mycelial growth in *Fusarium graminearum* has also been reported by Ribas et al. [65]. These mechanisms are potential ways our synthesized compounds could exhibit antimicrobial activity against tested bacteria and fungi.

Generally, it was observed in this study that the tested compounds had weaker antifungal activity compared to antibacterial activity (Table 3 and Table 4). This difference in sensitivity between bacteria and fungi towards the tested substances is not surprising, considering that Gram-positive bacteria have walls mainly consisting of mureins and teichoic acids. In contrast, Gram-negative bacteria have walls consisting of lipopolysaccharides and lipopoliproteins. On the other hand, fungal cell walls are complex structures made up of chitin, glucan, mannan, and diaminopimelic acid [66,67]. This difference between fungi and bacteria is why fungi have greater resistance.

The MIC values of individual compounds are linked to the length of the alkyl chain substituted at the *N* position of imidazolium cations. Generally, antibacterial activities are higher starting from molecular masses higher than 4 kDa (**3c**–**3f**) compared to lower molecular masses of PILs **3a** and **3b** that possessed lower MIC values. The findings suggest that the growth inhibition activity of ILs and PILs is tightly related to the alkyl side chain length. This result indicates that a long alkyl chain is a necessary structural component for potent antimicrobial activity. These results are consistent with previously published data on pyrrolidinium-based ILs and similar imidazolium-type ILs and PILs [29,68,69,70]. The same tendency with longer alkyl chains resulting in lower MIC values for IL monomers and PILs is shown by Zheng et al. In addition, the antimicrobial potency is compared to the charge density of imidazolium cations, indicating that bis-imidazolium ILs and PILs possess a better efficiency as antimicrobial agents than their mono-imidazolium analogues. The same group reported poor antimicrobial properties for PIL membranes prepared by photo-crosslinking of 1-octyl-3-vinylimidazolium bromide and 1-dodecyl-3-vinylimidazolium bromide with styrene and acrylonitrile [48]. Pyrrolidinium-based ILs and PILs exhibit similar trends with enhancing antimicrobial activity with the increase of the alkyl chain length, while PILs exhibit lower MIC values than their corresponding IL monomers [71]. 

From an application standpoint, flexible polymer membranes offer significant advantages over compounds that lack an exact spatial arrangement, which is the case for viscous ionic liquids (ILs) and solid polymeric ionic liquids (PILs). Moreover, the electrospinning technique provides an excellent opportunity to adjust the resulting fibers’ porosity, morphology, and thickness, serving as a great platform to design novel materials with tailored characteristics suitable for a wide range of applications, including filtration and medical settings. 

## 4. Conclusions

In this study, a broad structural diversity originated from the tunable nature of ILs and PILs, facile functionalization, and biological relevance of imidazoles coupled with the electrospinning technique were exploited for the preparation of novel polymeric ionic liquid-based membranes. A thorough synthetic route of a series of imidazolium-based ionic liquids with varying alkyl chain lengths, their polymers, and electrospun fiber preparation is provided. The experimental results showed that 1-dodecyl-3-vinylimidazolium bromide and its polymeric equivalent and fibers have the highest potential for inhibiting the growth of multispecies microorganisms with exceptionally low minimum inhibitory concentration (MIC) values comparable to the frequently used traditional antibiotics. This research strives to offer innovative antimicrobial strategies for creating materials with desired and adjustable chemical compositions, surfaces, and biological properties for addressing the increasing challenge of bacterial resistance.

As this area demands advanced solutions, our primary motivation is the development of antimicrobial performances born from the intrinsic polymer itself, avoiding the addition of active agents (nano-metals, essential oils, etc.) and accordingly preventing leakage from the application site, evaporation during the electrospinning process, and minimizing potential environmental impact. The resulting membranes exhibit similar MIC values as the initial polymers, opening up new approaches toward designer membranes and underscoring their potential in multiple fields where antimicrobial properties are crucial.

To fully understand the biomedical potential of the synthesized compounds, it is imperative to conduct further experiments. Specifically, it is necessary to verify their activity and reaction mechanism towards microorganisms, with a particular focus on ([C_12_VIm][Br]) and their corresponding polymers Poly([C_12_VIm][Br]) and Poly([C_12_VIm][Br]) electrospun fibers. These findings will yield crucial insights that could pave the way for pharmaceutical breakthroughs.

## Figures and Tables

**Table 1:** Chemical structures and abbreviations of synthesized ILs and PILs.

ILs/PILs	Chemical Structure	Abbreviation
1-ethyl-3-vinylimidazolium bromide	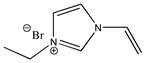	[C_2_VIm][Br] **2a**
1-butyl-3-vinylimidazolium bromide	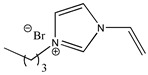	[C_4_VIm][Br] **2b**
1-hexyl-3-vinylimidazolium bromide	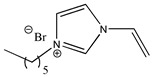	[C_6_VIm][Br] **2c**
1-octyl-3-vinylimidazolium bromide	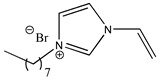	[C_8_VIm][Br] **2d**
1-decyl-3-vinylimidazolium bromide	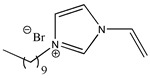	[C_10_VIm][Br] **2e**
1-dodecyl-3-vinylimidazolium bromide	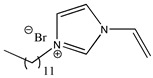	[C_12_VIm][Br] **2f**
Poly(1-ethyl-3-vinylimidazolium bromide)	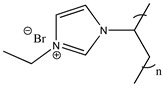	Poly([C_2_VIm][Br]) **3a**
Poly(1-butyl-3-vinylimidazolium bromide)	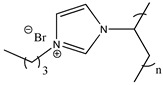	Poly([C_4_VIm][Br]) **3b**
Poly(1-hexyl-3-vinylimidazolium bromide)	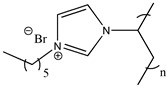	Poly([C_6_VIm][Br]) **3c**
Poly(1-octyl-3-vinylimidazolium bromide)	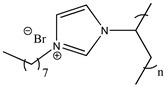	Poly([C_8_VIm][Br]) **3d**
Poly(1-decyl-3-vinylimidazolium bromide)	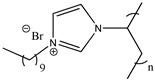	Poly([C_10_VIm][Br]) **3e**
Poly(1-dodecyl-3-vinylimidazolium bromide)	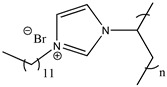	Poly([C_12_VIm][Br]) **3f**

**Table 2:** Thermal properties of 1-alkyl-3-vinylimidazolium bromide and poly(1-alkyl-3-vinylimidazolium bromide) were obtained from DSC.

ILs/PILs	*T*_g_/°C	*T*_m_/°C	*T*_c_/°C
[C_2_VIm][Br]	-	52.26 [50]	34.50 [50]
[C_4_VIm][Br]	−41.87 [50]	-	-
[C_6_VIm][Br]	−61 [50]	-	-
[C_8_VIm][Br]	−54.76 [50]	-	-
[C_10_VIm][Br]	−62.70 [50]	-	-
[C_12_VIm][Br]	-	39.51 [50]	1.83 [50]
Poly([C_2_VIm][Br])	-	75.75	52.66
Poly([C_4_VIm][Br])	100.80	-	-
Poly([C_6_VIm][Br])	175.04	242.98	-
Poly([C_8_VIm][Br])	-	-	-
Poly([C_10_VIm][Br])	104.56	180.65	-
Poly([C_12_VIm][Br])	176.08	-	-

**Table 3:** Antibacterial activities of imidazolium-based ILs, corresponding polymers, and electrospun fibers are measured as MIC (MIC values for Streptomycin and Doxycycline are also listed).

Bacteria	*Staphylococcus aureus*	*Bacillus subtilis*	*Klebsiella pneumoniae*	*Escherichia coli*	*Proteus mirabilis*
Tested Compounds and Fibers			MIC Values (mg/mL)		
[C_2_VIm][Br]	0.34	0.17	0.34	0.68	0.34
[C_4_VIm][Br]	3.31	0.825	1.65	3.31	1.65
[C_6_VIm][Br]	1.05	0.525	1.05	1.05	1.055
[C_8_VIm][Br]	0.366	0.09	0.366	0.732	0.366
[C_10_VIm][Br]	0.52	0.026	0.052	0.105	0.105
[C_12_VIm][Br]	0.005	0.001	0.005	0.01	0.01
Poly([C_2_VIm][Br])	0.79	0.39	0.79	0.79	0.79
Poly([C_4_VIm][Br])	0.747	0.186	0.373	0.747	0.33
Poly([C_6_VIm][Br])	0.165	0.041	0.082	0.165	0.082
Poly([C_8_VIm][Br])	0.74	0.186	0.372	0.745	0.372
Poly([C_10_VIm][Br])	0.64	0.32	0.64	0.64	0.64
Poly([C_12_VIm][Br])	0.09	0.022	0.045	0.09	0.09
Poly([C_10_VIm][Br]) fibers	0.83	0.42	0.83	0.83	1.66
Poly([C_12_VIm][Br]) fibers	0.015	0.06	0.06	0.03	0.11
Streptomycin	0.031	0.016	0.031	0.062	0.062
Doxycycline	0.0004	0.0019	0.004	0.0156	0.0156

**Table 4:** Antifungal activities of imidazolium-based ILs, corresponding PILs, and electrospun fibers are measured as MIC (MIC values for Ketoconazole and Fluconazole are also listed).

Fungi	*Aspergillus niger*	*Aspergillus flavus*	*Penicillium italicum*	*Mucor mucedo*	*Trichophyton mentagrophytes*
Tested Compounds and Fibers			MIC Values (mg/mL)		
[C_2_VIm][Br]	5.47	10.95	2.74	10.95	0.68
[C_4_VIm][Br]	13.23	26.46	26.45	26.46	0.08
[C_6_VIm][Br]	8.39	8.39	8.39	8.39	0.52
[C_8_VIm][Br]	1.465	1.465	1.465	0.732	0.183
[C_10_VIm][Br]	0.85	0.425	0.21	1.71	0.105
[C_12_VIm][Br]	0.01	0.02	0.02	0.041	0.01
Poly([C_2_VIm][Br])	12.76	12.76	12.76	1.276	0.79
Poly([C_4_VIm][Br])	5.98	5.98	1.495	5.98	0.186
Poly([C_6_VIm][Br])	0.66	0.66	0.66	2.63	0.33
Poly([C_8_VIm][Br])	2.99	1.49	1.49	2.99	0.74
Poly([C_10_VIm][Br])	5.15	2.06	2.06	1.03	0.64
Poly([C_12_VIm][Br])	0.72	0.36	0.72	2.91	0.09
Poly([C_10_VIm][Br]) fibers	1.66	1.66	1.66	0.83	0.83
Poly([C_12_VIm][Br]) fibers	0.46	0.46	0.06	0.12	0.06
Ketoconazole	0.07	0.31	0.15	0.25	0.15
Fluconazole	0.5	1	1	1	0.25

## Data Availability

Data are contained within the article.

## Supplementary Materials

The following supporting information can be downloaded at: https://www.mdpi.com/article/10.3390/polym16152094/s1. Table S1: Purity of the applied chemicals and reagents; Figure S1: (a) ^1^H and (b) ^13^C NMR spectra of the 1-ethyl-3-vinylimidazolium bromide **2a**; Figure S2: (a) ^1^H and (b) ^13^C NMR spectra for synthesized 1-butyl-3-vinylimidazolium bromide **2b**; Figure S3: (a) ^1^H and (b) ^13^C NMR spectra of the 1-hexyl-3-vinylimidazolium bromide **2c**; Figure S4: (a) ^1^H and (b) ^13^C NMR spectra of the 1-octyl-3-vinylimidazolium bromide **2d**; Figure S5: (a) ^1^H and (b) ^13^C NMR spectra of the 1-decyl-3-vinylimidazolium bromide **2e**; Figure S6: (a) ^1^H and (b) ^13^C NMR spectra of the 1-dodecyl-3-vinylimidazolium bromide **2f**; Figure S7: IR spectra of 1-ethyl-3-vinylimidazolium bromide **2a**; Figure S8: IR spectra of 1-butyl-vinylimidazolium bromide **2b**; Figure S9: IR spectra of 1-hexyl-3-vinylimidazolium bromide **2c**; Figure S10: IR spectra of 1-octyl-3-vinylimidazolium bromide **2d**; Figure S11: IR spectra of 1-decyl-3-vinylimidazolium bromide **2e**; Figure S12: IR spectra of 1-dodecyl-vinylimidazolium bromide **2f**; Figure S13: IR spectra of Poly(1-ethyl-3-vinylimidazolium bromide) **3a**; Figure S14: IR spectra of Poly(1-butyl-3-vinylimidazolium bromide) **3b**; Figure S15: IR spectra of Poly(1-hexyl-3-vinylimidazolium bromide) **3c**; Figure S16: IR spectra of Poly(1-octyl-3-vinylimidazolium bromide) **3d**; Figure S17: IR spectra of Poly(1-decyl-3-vinylimidazolium bromide) **3e**; Figure S18: IR spectra of Poly(1-dodecyl-3-vinylimidazolium bromide) **3f**; Figure S19: IR spectra of Poly(1-decyl-3-vinylimidazolium bromide) electrospun fibers; Figure S20: IR spectra of Poly(1-dodecyl-3-vinylimidazolium bromide) electrospun fibers; Figure S21: MS spectrum of Poly(1-ethyl-3-vinylimidazolium bromide) **3a**; Figure S22: MS spectrum of Poly(1-butyl-3-vinylimidazolium bromide) **3b**; Figure S23: MS spectrum of Poly(1-hexyl-3-vinylimidazolium bromide) **3c**; Figure S24: MS spectrum of Poly(1-octyl-3-vinylimidazolium bromide) **3d**; Figure S25: MS spectrum of Poly(1-decyl-3-vinylimidazolium bromide) **3e** (0.1% AIBN); Figure S26: MS spectrum of Poly(1-decyl-3-vinylimidazolium bromide) **3e** (0.5% AIBN); Figure S27: MS spectrum of Poly(1-decyl-3-vinylimidazolium bromide) **3e** (1% AIBN); Figure S28: MS spectrum of Poly(1-dodecyl-3-vinylimidazolium bromide) **3f** 1% AIBN; Figure S29: SEM micrograph of Poly([C10VIm][Br]) fibers at magnification: X1000; Figure S30: SEM micrograph of Poly([C_12_VIm][Br]) fibers at magnification: X500.
