# Supplementary material for: From Synthesis to Functionality: Tailored Ionic Liquid-Based Electrospun Fibers with Superior Antimicrobial Properties
Source: Polymers (Basel). 2024 Jul 23;16(15):2094. doi: 10.3390/polym16152094 (PMC11314316; doi:10.3390/polym16152094)
Supplement: Supplementary file 1 [file polymers-16-02094-s001.zip › polymers-3050869-supplementary.pdf]

Supplementary Material

From Synthesis to Functionality: Tailored Ionic Liquid based Electrospun fibers with Superior Antimicrobial Properties

Sanja Rackov[1,*], Branka Pilić[1*], Nenad Janković[2], Marijana Kosanić[3], Marijana Petković[4] and Milan Vraneš[5]

[1]*Faculty of Technology Novi Sad, Department of Materials Engineering, University of Novi Sad, Bulevar cara Lazara 1, 21000 Novi Sad, Serbia, sanja.rackov@uns.ac.rs, brapi@uns.ac.rs*

[2]*Institute for Information Technologies, University of Kragujevac, Radoja Domanovića 12, 34000 Kragujevac, Serbia, nenad.jankovic@uni.kg.ac.rs*

[3]*Faculty of Science, Department of Biology and Ecology, University of Kragujevac, Radoja Domanovića 12, 34000 Kragujevac, Serbia, marijana.kosanic@pmf.kg.ac.rs*

[4]*Department of Atomic Physics, "Vinča" Institute of Nuclear Sciences - National Institute of the Republic of Serbia, University of Belgrade, Belgrade, Serbia, marijanapetkovic@vin.bg.ac.rs*

[5]*Faculty of Sciences, Department of Chemistry, Biochemistry and Environmental Protection, University of Novi Sad, Trg Dositeja Obradovića 3, 21000 Novi Sad, Serbia, milan.vranes@dh.uns.ac.rs*

Table S1. Purity of the applied chemicals and reagents.

Chemical Name	CAS number	Source	Mass Fraction Purity	Purification Method
1-vinylimidazole	1072-63-5	Sigma-Aldrich	≥ 0.99**	none
Bromoethane	74-96-4	Sigma-Aldrich	0.98**	none
1-Bromobutane	109-65-9	Sigma-Aldrich	≥ 0.99**	none
1-Bromohexane	111-25-1	Sigma-Aldrich	≥ 0.98**	none
1-Bromooctane	111-83-1	Sigma-Aldrich	0.99**	none
1-Bromodecane	112-29-8	Acros-Organics	0.98**	none
1-Bromododecane	143-15-7	Sigma-Aldrich	0.98**	none
Ethyl acetate	141-78-6	Fischer-Chemical	≥ 0.998**	none
Chloroform	67-66-3	Sigma-Aldrich	0.998**	none
1-ethyl-3-vinylimidazolium bromide	34311-88-1	Synthesis	0.96*	Liquid-liquid extraction, rotary evaporation followed by vacuum
1-butyl-3-vinylimidazolium bromide	34311-90-5	Synthesis	0.97*	Rotary evaporation followed by vacuum
1-hexyl-3-vinylimidazolium bromide	74921-79-2	Synthesis	0.98*	Rotary evaporation followed by vacuum
1-octyl-3-vinylimidazolium bromide	349148-76-1	Synthesis	0.95*	Rotary evaporation followed by vacuum
1-decyl-3-vinylimidazolium bromide	349148-78-3	Synthesis	0.96*	Rotary evaporation followed by vacuum
1-dodecyl-3-vinylimidazolium bromide	155085-25-9	Synthesis	0.97*	Rotary evaporation followed by vacuum

*Provided by the supplier.

**Determined by NMR measurement.

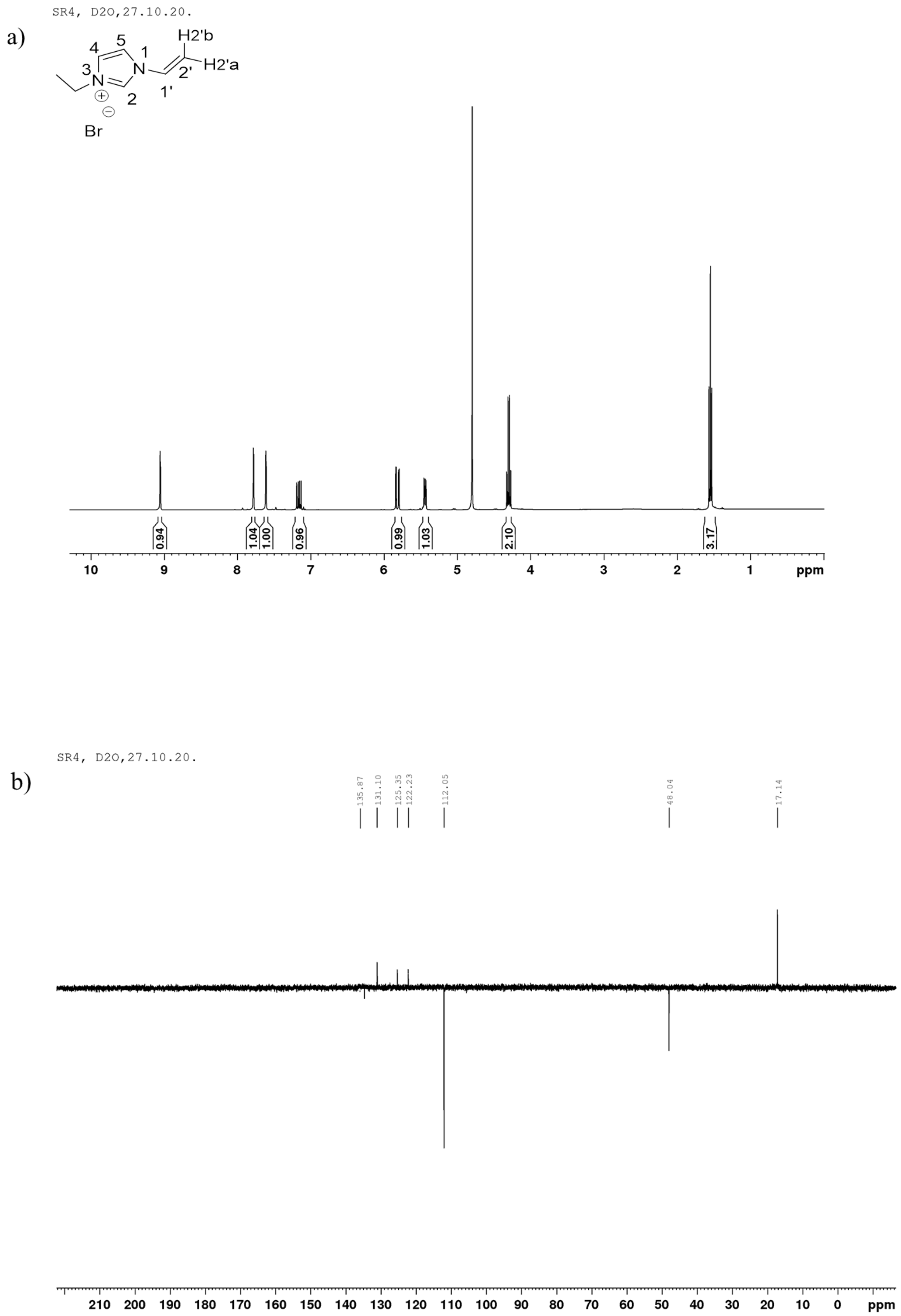

Figure S1. a) ^{1}H and b) ^{13}C NMR spectra of the 1-ethyl-3-vinylimidazolium bromide **2a**

^{1}H NMR (D_2O, ppm): 1.55 (t, 3H, $J_{CH2,CH3}$ = 7.4 Hz, C*H*$_3$), 4.30 (q, 2H, $J_{CH2,CH3}$= 7.4 Hz, C*H*$_2$), 5.44 (dd, 1H, $J_{2a',1'}$ = 8.7 Hz, $J_{2a',2b'}$ = 2.8 Hz, H-2a'), 5.82 (dd, 1H, $J_{2b',1'}$ = 15.6 Hz, $J_{2a',2b'}$ = 2.8 Hz, H-2b'), 7.16 (dd, 1H, $J_{2a',1'}$ = 8.7 Hz, $J_{2b',1'}$ = 15.6 Hz, H-1'), 7.61 (d, 1H, $J_{4,5}$ = 1.9 Hz, H-4), 7.78 (d, 1H, $J_{4,5}$ = 1.9 Hz, H-5), 9.06 (bs, 1H, H-2).

^{13}C NMR (D_2O, ppm): 17.1 (CH_3), 48.0 (CH_2), 112.1 (CH_2-2′), 122.2 (C-5), 125.4 (C-4), 131.1 (C-1′), 135.9 (C-2).

a)

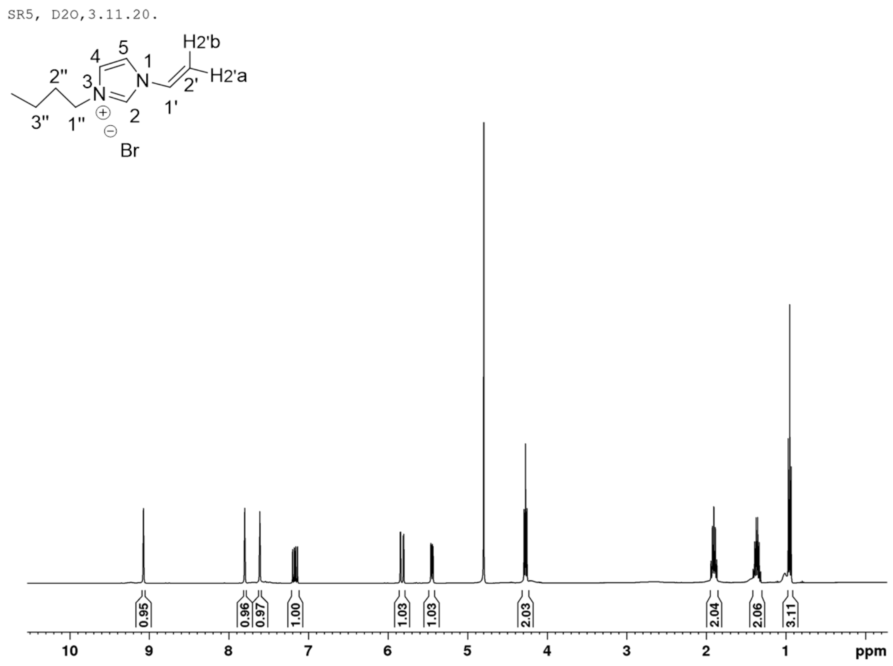

b)

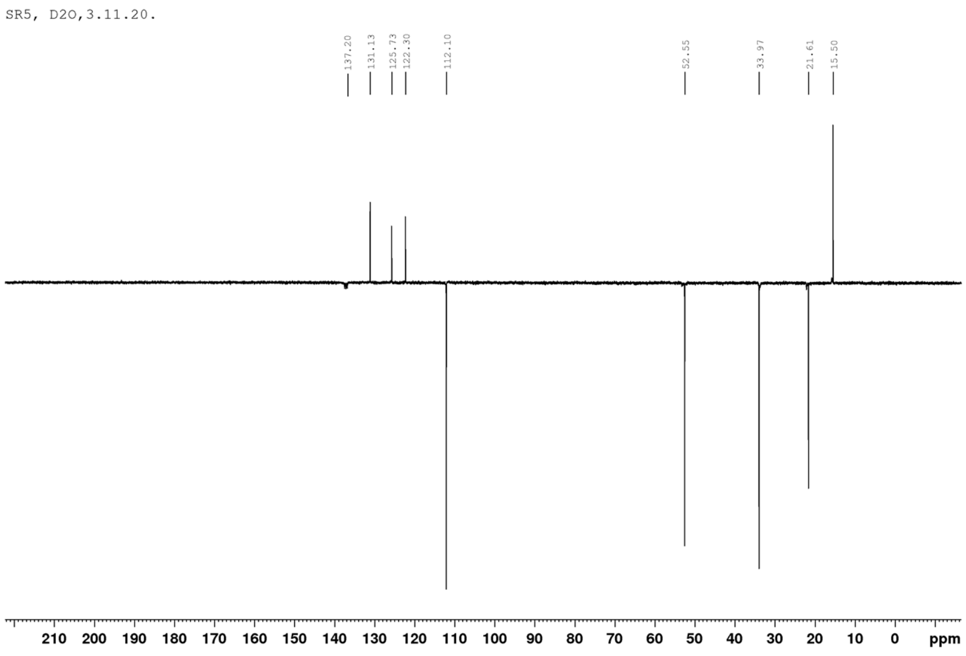

Figure S2. a) ^1H and b) ^{13}C NMR spectra for synthesized 1-butyl-3-vinylimidazolium bromide **2b**

^1H NMR (D_2O, ppm): 0.95 (t, 3H, J = 7.5 Hz, CH_3), 1.36 (m, 2H, CH_2-3″), 1.90 (m, 2H, CH_2-2″), 4.27 (t, 2H, J = 7.4 Hz, CH_2-1″), 5.45 (dd, 1H, $J_{2a',1'}$ = 8.7 Hz, $J_{2a',2b'}$ = 2.8 Hz, H-2a′), 5.82 (dd, 1H,

$J_{2b',1'}$ = 15.6 Hz, $J_{2a',2b'}$ = 2.8 Hz, H-2b′), 7.17 (dd, 1H, $J_{2a',1'}$ = 8.7 Hz, $J_{2b',1'}$ = 15.6 Hz, H-1′), 7.61 (t, 1H, $J_{4,5}$ = 1.9 Hz, $J_{2,4}$ = 1.9 Hz, H-4), 7.80 (t, 1H, $J_{4,5}$ = 1.9 Hz, $J_{2,5}$ = 1.9 Hz, H-5), 9.07 (bs, 1H, H-2).

^{13}C NMR (D_2O, ppm): 15.5 (CH_3), 21.6 (CH_2-3″), 33.9 (CH_2-2″), 52.6 (CH_2-1″), 112.1 (CH_2-2′), 122.3 (C-5), 125.7 (C-4), 131.1 (C-1′), 137.2 (C-2).

a)

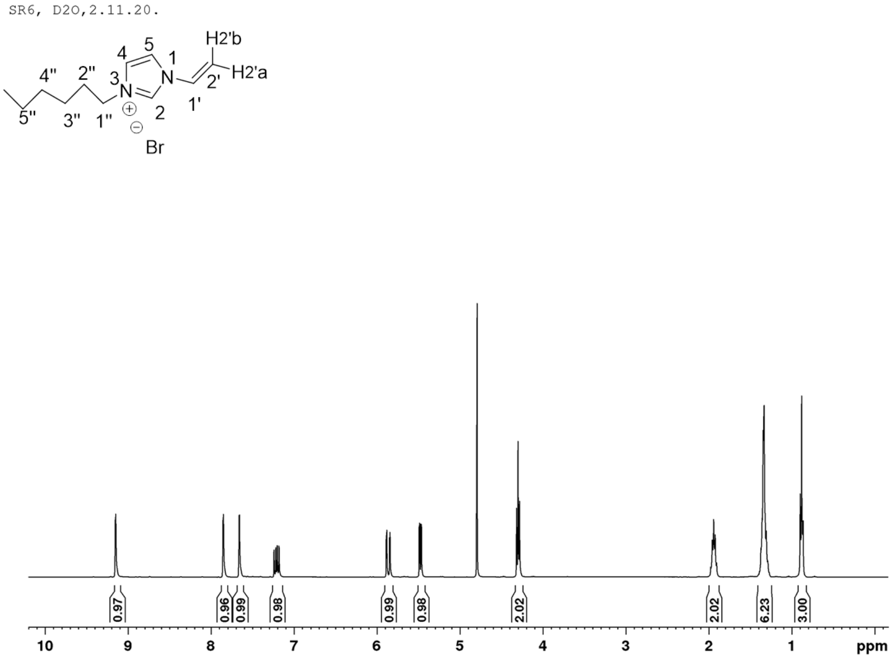

b)

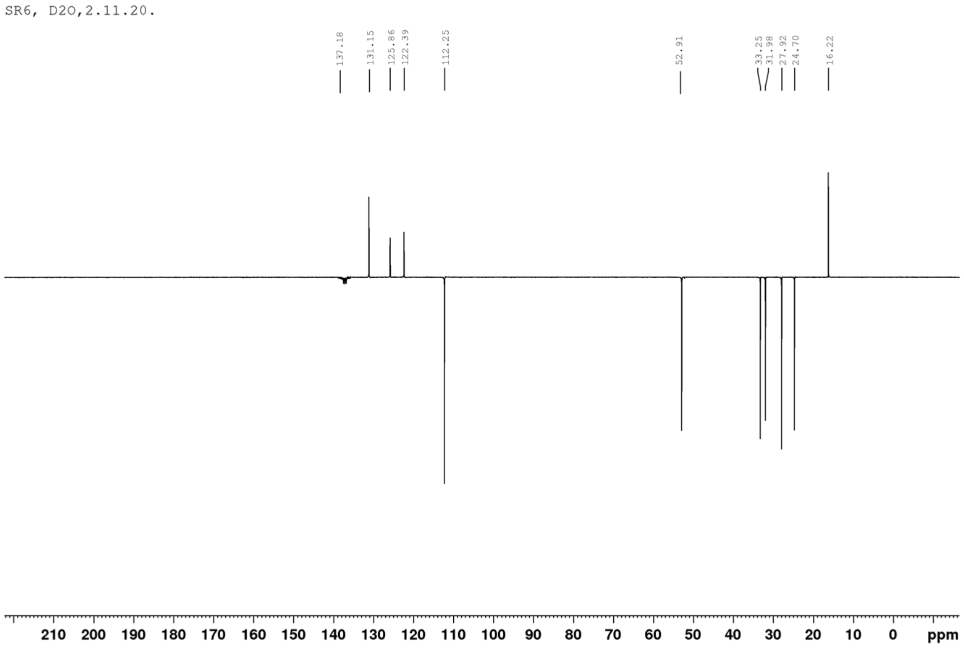

Figure S3. a) ^{1}H and b) ^{13}C NMR spectraof the 1-hexyl-3-vinylimidazolium bromide **2c**

^{1}H NMR (D_2O, ppm): 0.88 (t, 3H, J = 7.1 Hz, CH_3), 1.26-1.40 (m, 6H, CH_2-3″, CH_2-4″, CH_2-5″), 1.94 (m, 2H, CH_2-2″), 4.30 (t, 2H, J = 7.1 Hz, CH_2-1″), 5.48 (dd, 1H, $J_{2a',1'}$ = 8.7 Hz, $J_{2a',2b'}$ = 2.8 Hz, H-2a′), 5.86 (dd, 1H, $J_{2b',1'}$ = 15.6 Hz, $J_{2a',2b'}$ = 2.8 Hz, H-2b′), 7.21 (dd, 1H, $J_{2a',1'}$ = 8.7 Hz, $J_{2b',1'}$ = 15.6 Hz, H-1′), 7.66 (d, 1H, $J_{4,5}$ = 1.9 Hz, H-4), 7.85 (d, 1H, $J_{4,5}$ = 1.9 Hz, H-5), 9.14 (bs, 1H, H-2).

^{13}C NMR (D_2O, ppm): 16.2 (CH_3), 24.7, 27.9, 33.3 (CH_2-3″, CH_2-4″, CH_2-5″), 31.9 (CH_2-2″), 52.9 (CH_2-1″), 112.3 (CH_2-2′), 122.4 (C-5), 125.9 (C-4), 131.2 (C-1′), 137.2 (C-2).

a)

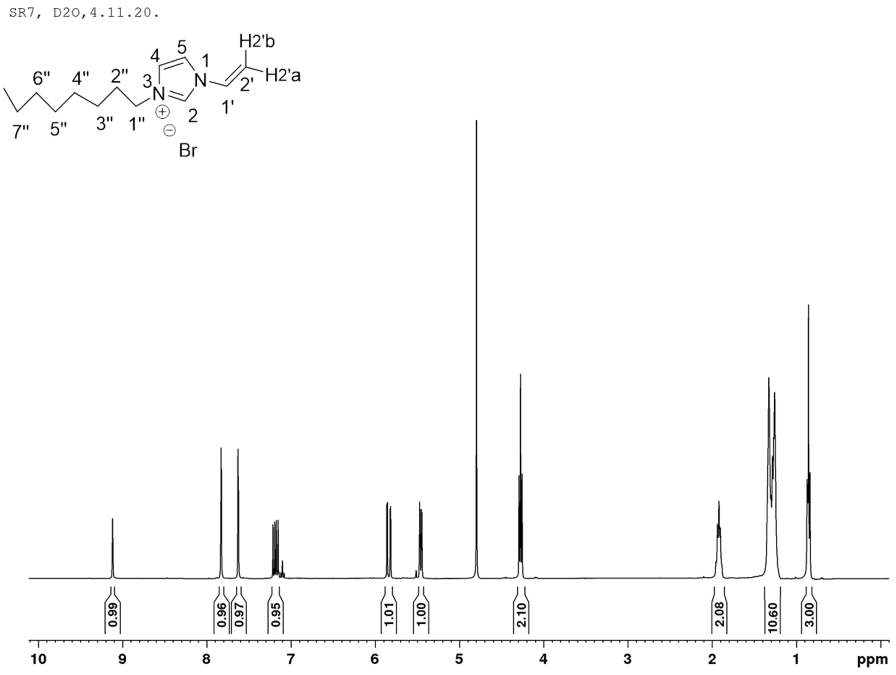

b)

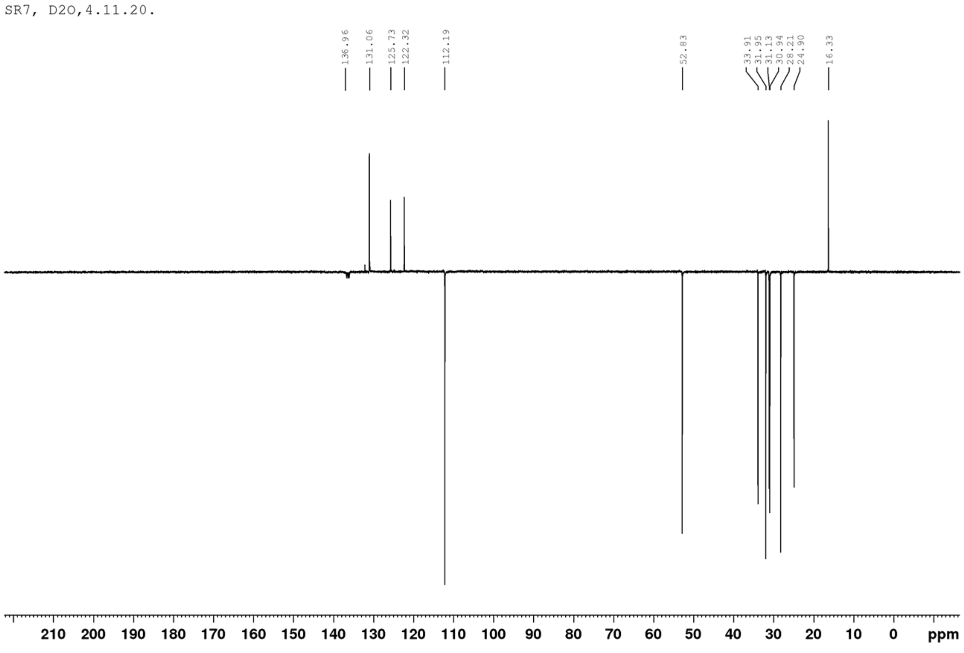

Figure S4. a) ^{1}H and b) ^{13}C NMR spectra of the 1-octyl-3-vinylimidazolium bromide **2d**

^{1}H NMR (D_2O, ppm): 0.85 (t, 3H, J = 7.1 Hz, J = 6.7 Hz, CH_3), 1.21-1.36 (m, 10H, CH_2-3″, CH_2-4″, CH_2-5″, CH_2-6″, CH_2-7″), 1.92 (m, 2H, CH_2-2″, 4.27, t, 2H, J = 7.1 Hz, CH_2-1″), 5.46 (dd, 1H, $J_{2a',1'}$ = 8.7 Hz, $J_{2a',2b'}$ = 2.8 Hz, H-2a′), 5.84 (dd, 1H, $J_{2b',1'}$ = 15.7 Hz, $J_{2a',2b'}$ = 2.8 Hz, H-2b′), 7.18 (dd, 1H, $J_{2a',1'}$ = 8.7 Hz, $J_{2b',1'}$ = 15.6 Hz, H-1′), 7.63 (d, 1H, $J_{4,5}$ = 1.9 Hz, H-4), 7.83 (d, 1H, $J_{4,5}$ = 1.9 Hz, H-5), 9.12 (bs, 1H, H-2).

^{13}C NMR (D_2O, ppm): 16.3 (CH_3), 24.9, 28.2, 30.9, 31.1, 33.9 (CH_2-3″, CH_2-4″, CH_2-5″ , CH_2-6″, CH_2-7″), 31.9 (CH_2-2″), 52.8 (CH_2-1″), 112.2 (CH_2-2′), 122.3 (C-5), 125.7 (C-4), 131.1 (C-1′), 136.9 (t, $J_{2,D}$= 34.4 Hz, C-2).

a)

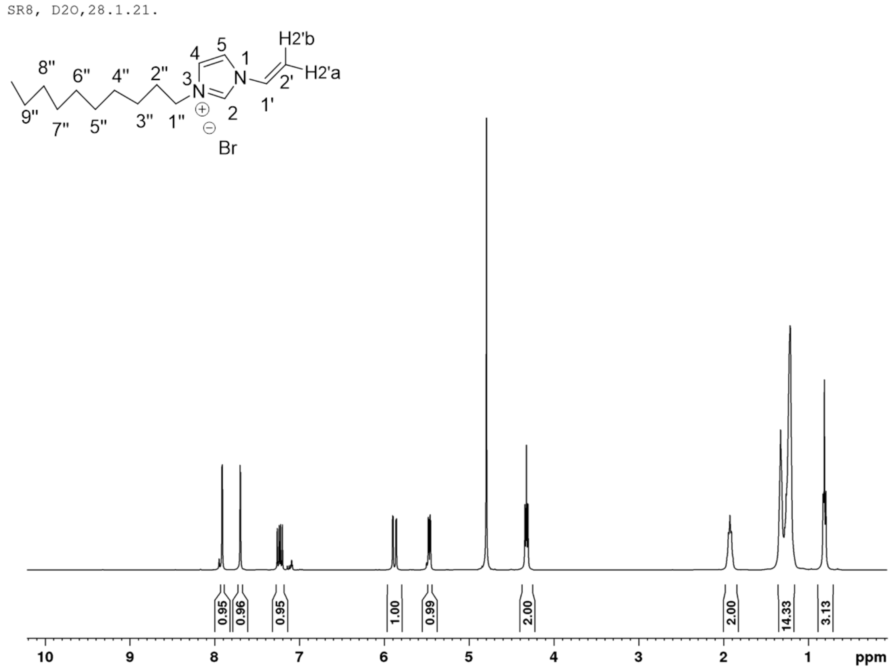

b)

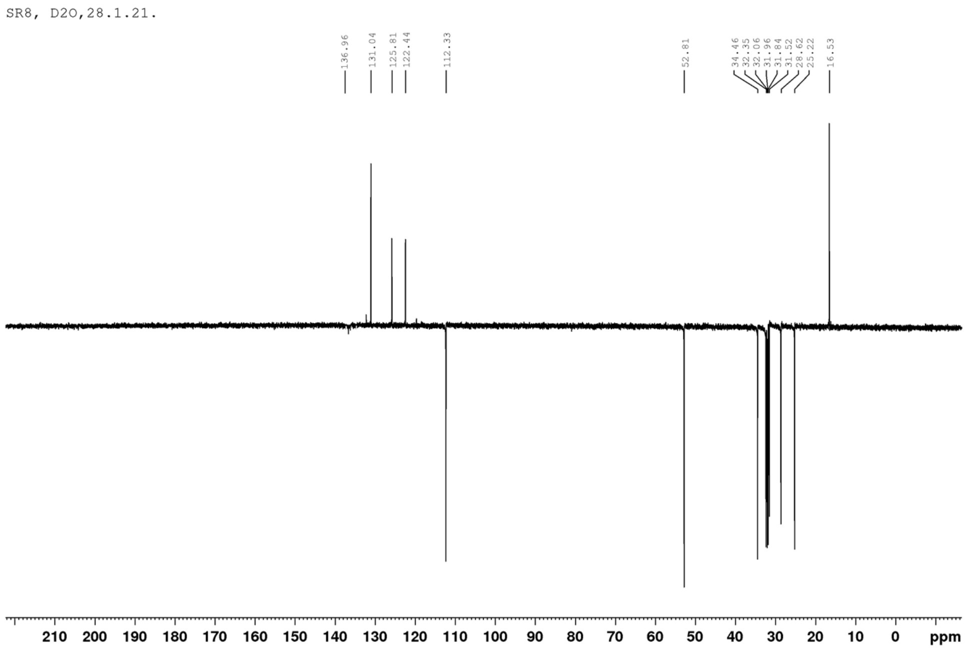

Figure S5. a) ^{1}H and b) ^{13}C NMR spectra of the 1-decyl-3-vinylimidazolium bromide **2e**

^{1}H NMR (D_2O, ppm): 0.80 (t, 3H, J = 6.9 Hz, J = 6.3 Hz, CH_3), 1.13-1.38 (m, 14H, CH_2-3″, CH_2-4″, CH_2-5″, CH_2-6″, CH_2-7″, CH_2-8″, CH_2-9″), 1.92 (m, 2H, CH_2-2″, 4.32, t, 2H, J = 7.3 Hz, CH_2-1″), 5.46 (dd, 1H, $J_{2a',1'}$ = 8.7 Hz, $J_{2a',2b'}$ = 2.8 Hz, H-2a′), 5.88 (dd, 1H, $J_{2b',1'}$ = 15.6 Hz, $J_{2a',2b'}$ = 2.8 Hz, H-2b′), 7.23 (dd, 1H, $J_{2a',1'}$ = 8.7 Hz, $J_{2b',1'}$ = 15.6 Hz, H-1′), 7.77 (d, 1H, $J_{4,5}$ = 2.0 Hz, H-4), 7.99 (d, 1H, $J_{4,5}$ = 2.0 Hz, H-5).

^{13}C NMR (D_2O, ppm): 16.5 (CH_3), 25.2, 28.6, 31.5, 31.8, 31.9, 32.1, 34.5 (CH_2-3″, CH_2-4″, CH_2-5″, CH_2-6″, CH_2-7″, CH_2-8″, CH_2-9″), 32.4 (CH_2-2″), 52.8 (CH_2-1″), 112.3 (CH_2-2′), 122.4 (C-5), 125.8 (C-4), 131.0 (C-1′), 136.9 (t, $J_{2,D}$ = 34.1 Hz, C-2).

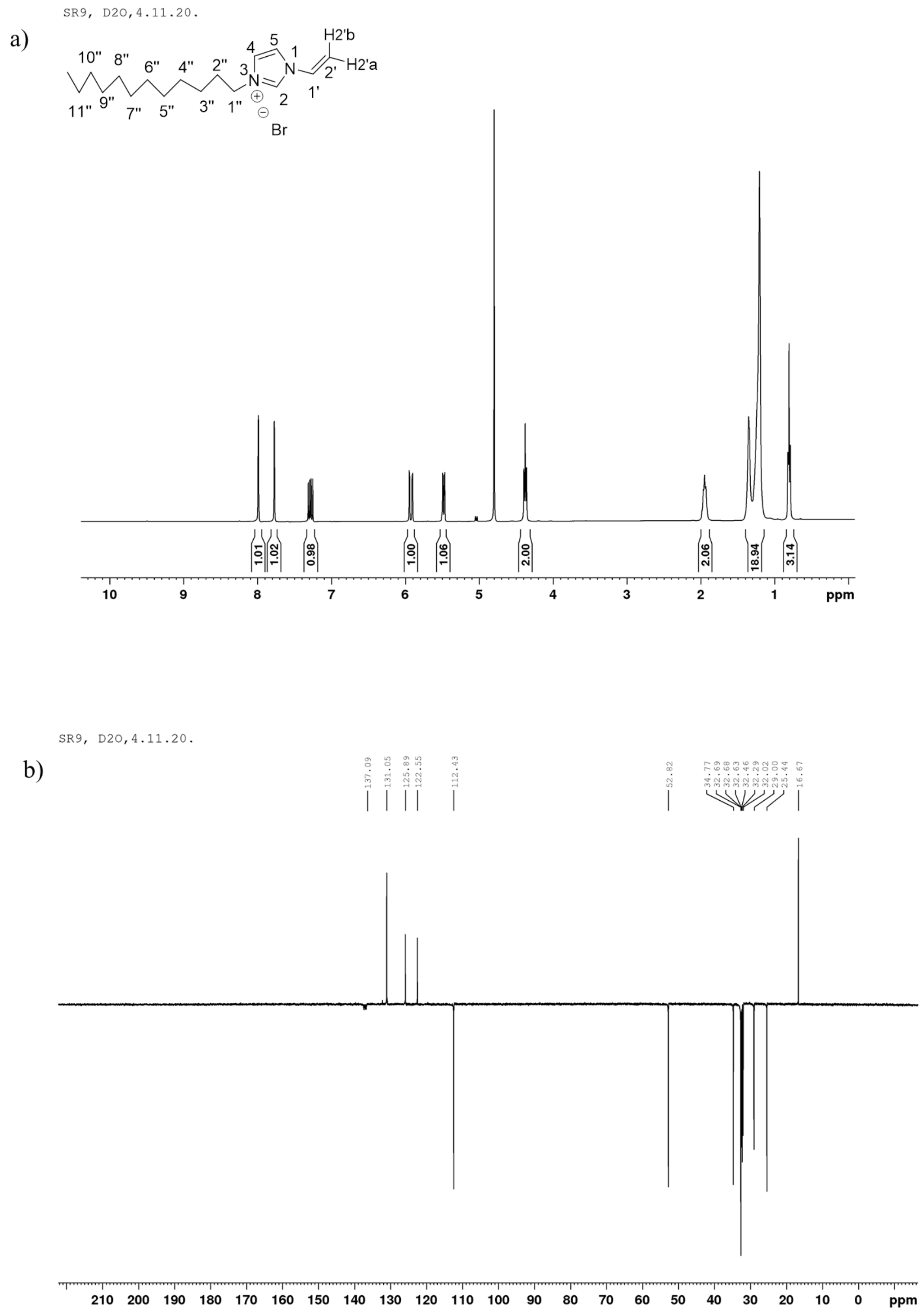

Figure S6. a) ^{1}H and b) ^{13}C NMR spectra of the 1-dodecyl-3-vinylimidazolium bromide **2f**

^{1}H NMR (D_2O, ppm): 0.80 (t, 3H, J = 6.9 Hz, J = 6.3 Hz, CH_3); 1.08-1.41 (m, 18H, CH_2-3″, CH_2-4″, CH_2-5″, CH_2-6″, CH_2-7″, CH_2-8″, CH_2-9″, CH_2-10″, CH_2-11″); 1.95 (m, 2H, CH_2-2″, 4.38, t, 2H, J = 7.3 Hz, CH_2-1″); 5.48 (dd, 1H, $J_{2a',1'}$ = 8.7 Hz, $J_{2a',2b'}$ = 2.8 Hz, H-2a′); 5.92 (dd, 1H, $J_{2b',1'}$ = 15.6 Hz, $J_{2a',2b'}$ = 2.8 Hz, H-2b′); 7.18 (dd, 1H, $J_{2a',1'}$ = 8.7 Hz, $J_{2b',1'}$ = 15.6 Hz, H-1′); 7.77 (d, 1H, $J_{4,5}$ = 1.9 Hz, H-4); 7.99 (d, 1H, $J_{4,5}$ = 1.9 Hz, H-5).

^{13}C NMR (D_2O, ppm): 16.7 (CH_3); 25.4, 29.0, 32.0, 32.3, 32.5, 32.6, 32.7, 32.7, 34.8 (CH_2-3″, CH_2-4″, CH_2-5″, CH_2-6″, CH_2-7″, CH_2-8″, CH_2-9″, CH_2-10″, CH_2-11″), 31.9 (CH_2-2″), 52.8 (CH_2-1″), 112.4 (CH_2-2′), 122.6 (C-5), 125.9 (C-4), 131.1 (C-1′), 137.1 (t, $J_{2,D}$ = 33.8 Hz, C-2).

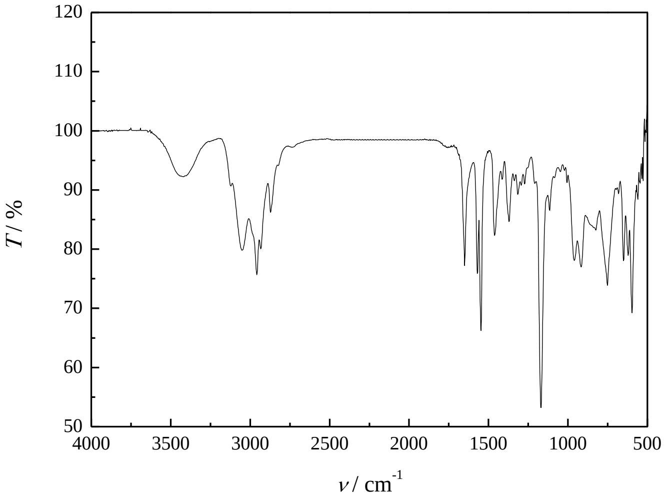

Figure S7. IR spectra of 1-ethyl-3-vinylimidazolium bromide **2a**

IR neat: 3120 cm^{-1} (sym. stretching ν HC((4)C(5)H); 3053 (sym. stretching *ν* of imidazole ring *ν* CH_3 HCH); 2958 cm^{-1}, 2931 cm^{-1} (asym. stretching *ν* C-H of -CH_3 and =CH_2); 2872 cm^{-1} (sym. stretching *ν* CH_3); 1651 cm^{-1} (stretching *ν* C=C); 1570 cm^{-1}, 1546 cm^{-1} (in-plane *ν* of imidazolium ring); 1462 cm^{-1}, 1413 cm^{-1} (C-N stretching of imidazole ring); 1338 cm^{-1} (in-plane asym. stretching *ν* of imidazole ring, CCCC stretching); 1170 cm^{-1} (sceletal *ν* of imidazolium ring); 916 cm^{-1} (out-of-plane bending *ν* CH=CH_2); 752 cm^{-1} (out-of-plane C-H bending *ν* of imidazole ring); 650 cm^{-1} (C2-N-C5 bending *ν* of imidazole ring); 621 cm^{-1}, 596 cm^{-1} (CCC in-plane ring bending).

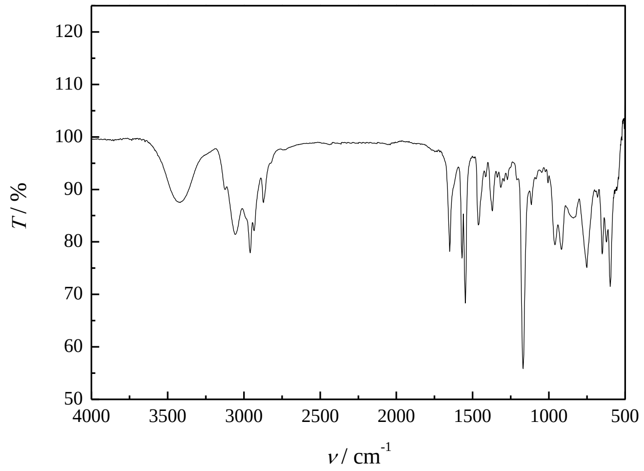

Figure S8. IR spectra of 1-butyl-vinylimidazolium bromide **2b**

IR neat: 3120 cm^{-1} (sym. stretching ν HC((4)C(5)H); 3051 cm^{-1} (sym. stretching *ν* of imidazole ring *ν* CH_3 HCH); 2958 cm^{-1}, 2933 cm^{-1} (asym. stretching *ν* C-H of -CH_3 and =CH_2); 2866 cm^{-1} (sym. stretching *ν* CH_3); 1651 cm^{-1} (stretching *ν* C=C); 1570 cm^{-1}, 1546 cm^{-1} (in-plane *ν* of

imidazolium ring); 1462 cm^{-1}, 1413 cm^{-1} (C-N stretching of imidazole ring); 1336 cm^{-1} (in-plane asym. stretching ν of imidazole ring, CCCC stretching); 1170 cm^{-1} (sceletal ν of imidazolium ring); 916 cm^{-1} (out-of-plane bending ν $CH=CH_2$); 752 cm^{-1} (out-of-plane C-H bending ν of imidazole ring); 650 cm^{-1} (C2-N-C5 bending ν of imidazole ring); 621 cm^{-1}, 596 cm^{-1} (CCC in-plane ring bending).

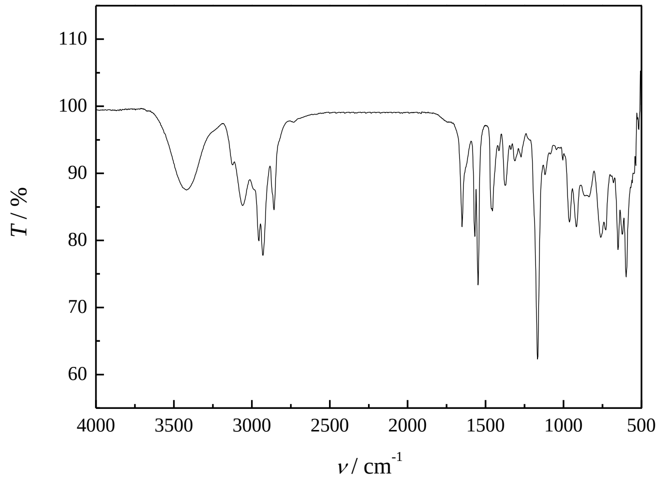

Figure S9. IR spectra of 1-hexyl-3-vinylimidazolium bromide **2c**

IR neat: 3118 cm^{-1} (sym. stretching ν HC((4)C(5)H); 3051 cm^{-1} (sym. stretching ν of imidazole ring ν CH_3 HCH); 2954 cm^{-1}, 2927 cm^{-1} (asym. stretching ν C-H of $-CH_3$ and $=CH_2$); 2856 cm^{-1} (sym. stretching ν CH_3); 1651 cm^{-1} (stretching ν C=C); 1570 cm^{-1}, 1546 cm^{-1} (in-plane ν of imidazolium ring); 1454 cm^{-1}, 1413 cm^{-1} (C-N stretching of imidazole ring); 1336 (in-plane asym. stretching ν of imidazole ring, CCCC stretching); 1166 cm^{-1} (sceletal ν of imidazolium ring); 918 cm^{-1} (out-of-plane bending ν $CH=CH_2$); 763 cm^{-1} (out-of-plane C-H bending ν of imidazole ring); 650 cm^{-1} (C2-N-C5 bending ν of imidazole ring); 624 cm^{-1}, 597 cm^{-1} (CCC in-plane ring bending).

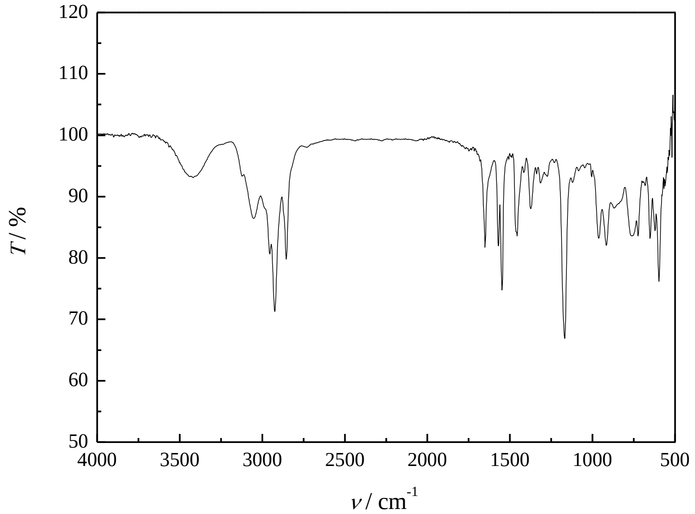

Figure S10. IR spectra of 1-octyl-3-vinylimidazolium bromide **2d**

IR neat: 3122 cm^{-1} (sym. stretching ν HC((4)C(5)H); 3053 cm^{-1} (sym. stretching ν of imidazole ring ν CH_3 HCH); 2954 cm^{-1}, 2924 cm^{-1} (asym. stretching ν C-H of -CH_3 and =CH_2); 2949 cm^{-1} (asym. stretching ν CH_2); 2854 cm^{-1} (sym. stretching ν CH_3); 1651 cm^{-1} (stretching ν C=C); 1570 cm^{-1}, 1546 cm^{-1} (in-plane ν of imidazolium ring); 1456 cm^{-1}, 1417 cm^{-1} (C-N stretching of imidazole ring); 1338 cm^{-1} (in-plane asym. stretching ν of imidazole ring, CCCC stretching); 1168 cm^{-1} (sceletal ν of imidazolium ring); 916 cm^{-1} (out-of-plane bending ν CH=CH_2); 759 cm^{-1} (out-of-plane C-H bending ν of imidazole ring); 650 cm^{-1} (C2-N-C5 bending ν of imidazole ring); 621 cm^{-1}, 597 cm^{-1} (CCC in-plane ring bending).

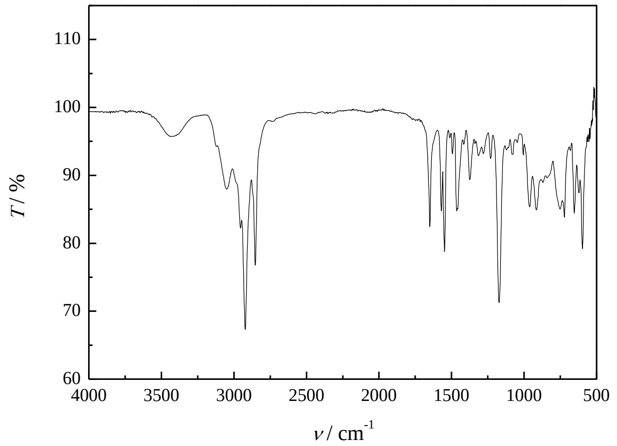

Figure S11. IR spectra of 1-decyl-3-vinylimidazolium bromide **2e**

IR neat: 3116 cm^{-1} (sym. stretching ν HC((4)C(5)H); 3049 cm^{-1} (sym. stretching ν of imidazole ring ν CH_3 HCH); 2954 cm^{-1}, 2922 cm^{-1} (asym. stretching ν C-H of -CH_3 and =CH_2); 2949 cm^{-1} (asym. stretching ν CH_2); 2852 cm^{-1} (sym. stretching ν CH_3); 1651 cm^{-1} (stretching ν C=C); 1570 cm^{-1}, 1548 cm^{-1} (in-plane ν of imidazolium ring); 1456 cm^{-1}, 1417 cm^{-1} (C-N stretching of imidazole ring); 1338 cm^{-1} (in-plane asym. stretching ν of imidazole ring, CCCC stretching); 1172 cm^{-1} (sceletal ν of imidazolium ring); 916 cm^{-1} (out-of-plane bending ν CH=CH_2); 754 cm^{-1} (out-of-plane C-H bending ν of imidazole ring); 651 cm^{-1} (C2-N-C5 bending ν of imidazole ring); 621 cm^{-1}, 599 cm^{-1} (CCC in-plane ring bending).

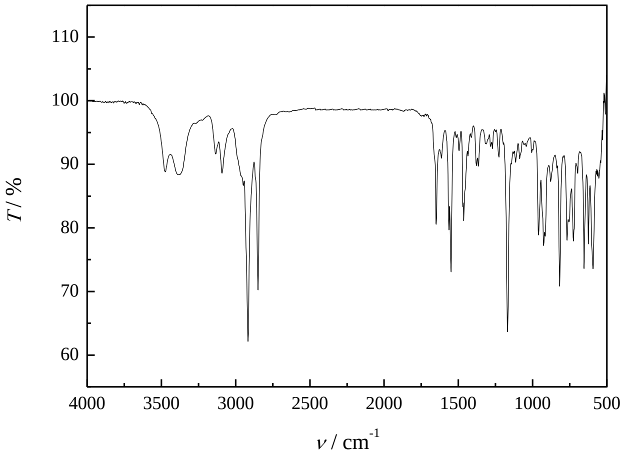

Figure S12. IR spectra of 1-dodecyl--vinylimidazolium bromide **2f**

IR neat: 3134 cm^{-1} (sym. stretching ν HC((4)C(5)H); 3088 cm^{-1} (sym. stretching *ν* of imidazole ring *ν* CH_3 HCH); 2949 cm^{-1}, 2916 cm^{-1} (asym. stretching *ν* C-H of -CH_3 and =CH_2); 2949 cm^{-1} (asym. stretching *ν* CH_2); 2848 cm^{-1}, 1379 cm^{-1} (sym. stretching *ν* CH_3); 1649 cm^{-1} (stretching *ν* C=C); 1564 cm^{-1}, 1550 cm^{-1} (in-plane *ν* of imidazolium ring); 1463 cm^{-1} (C-N stretching of imidazole ring); 1367 cm^{-1} (in-plane asym. stretching *ν* of imidazole ring, CCCC stretching); 1172 cm^{-1} (sceletal *ν* of imidazolium ring); 925 cm^{-1} (out-of-plane bending *ν* CH=CH_2); 767 cm^{-1} (out-of-plane C-H bending *ν* of imidazole ring); 653 cm^{-1} (C2-N-C5 bending *ν* of imidazole ring); 624 cm^{-1}, 592 cm^{-1} (CCC in-plane ring bending).

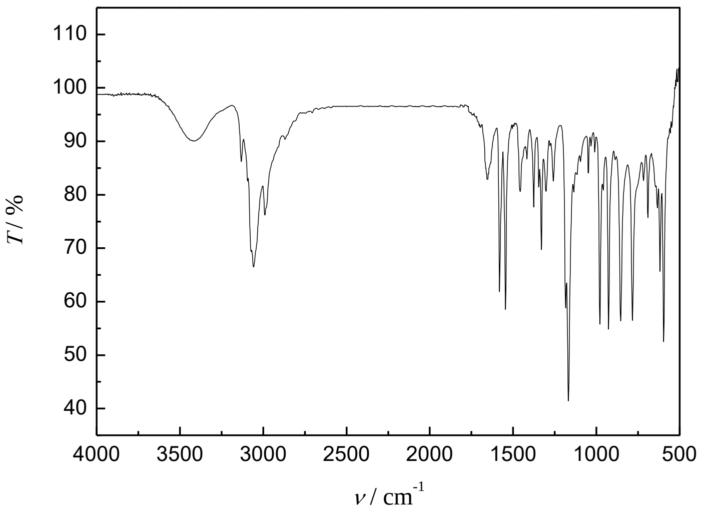

Figure S13. IR spectra of Poly(1-ethyl-3-vinylimidazolium bromide) **3a**

IR neat: 3130 cm^{-1} (sym. stretching ν HC((4)C(5)H); 3057 cm^{-1} (sym. stretching *ν* of imidazole ring *ν* CH_3 HCH); 2985 cm^{-1} (asym. stretching *ν* CH_2); 1660 cm^{-1} (stretching *ν* C=C); 1579 cm^{-1}, 1544 cm^{-1} (in-plane *ν* of imidazolium ring); 1458 cm^{-1}, 1415 cm^{-1} (C-N stretching of imidazole ring); 1328 cm^{-1} (in-plane asym. stretching *ν* of imidazole ring, CCCC stretching); 1168 cm^{-1} (sceletal *ν* of imidazolium ring); 783 cm^{-1} (out-of-plane C-H bending *ν* of imidazole ring) 690 cm^{-1} (C2-N-C5 bending *ν* of imidazole ring).

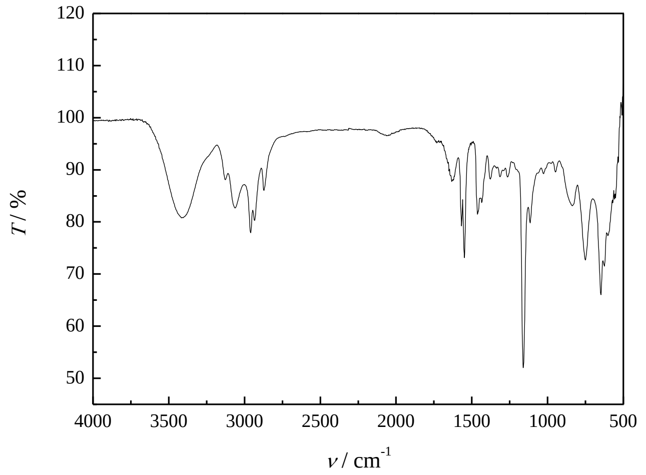

Figure S14. IR spectra of Poly(1-butyl-3-vinylimidazolium bromide) **3b**

IR neat: 3120 cm^{-1} (sym. stretching ν HC((4)C(5)H); 3053 cm^{-1} (sym. stretching *ν* of imidazole ring *ν* CH_3 HCH); 2962 cm^{-1} (asym. stretching *ν* C-H); 2865 cm^{-1} (sym. stretching *ν* CH_3); 1652 cm^{-1} (stretching *ν* C=C); 1565 cm^{-1}, 1539 cm^{-1} (in-plane *ν* of imidazolium ring);1463 cm^{-1} (C-N stretching of imidazole ring); 1172 cm^{-1} (sceletal *ν* of imidazolium ring); 913 cm^{-1} (out-of-plane bending *ν* CH=CH_2); 600 cm^{-1} (CCC in-plane ring bending).

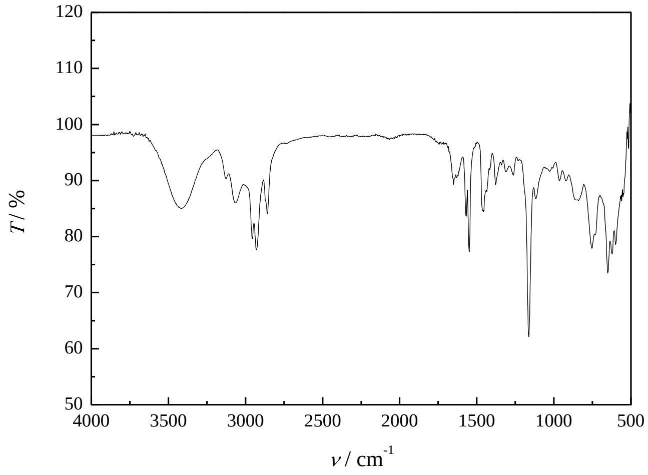

Figure S15. IR spectra of Poly(1-hexyl-3-vinylimidazolium bromide) **3c**

IR neat: 3129 cm^{-1} (sym. stretching ν HC((4)C(5)H); 3063 cm^{-1} (sym. stretching *ν* of imidazole ring *ν* CH_3 HCH); 2961 cm^{-1}, 2923 cm^{-1} (asym. stretching *ν* C-H); 2857 cm^{-1} (sym. stretching *ν* CH_3); 1646 cm^{-1} (stretching *ν* C=C); 1564 cm^{-1}, 1554 cm^{-1} (in-plane *ν* of imidazolium ring); 1460 (C-N stretching of imidazole ring); 1374 (in-plane asym. stretching *ν* of imidazole ring, CCCC stretching); 1156 cm^{-1} (sceletal *ν* of imidazolium ring); 747 cm^{-1} (out-of-plane C-H bending *ν* of imidazole ring); 655 cm^{-1} (C2-N-C5 bending *ν* of imidazole ring).

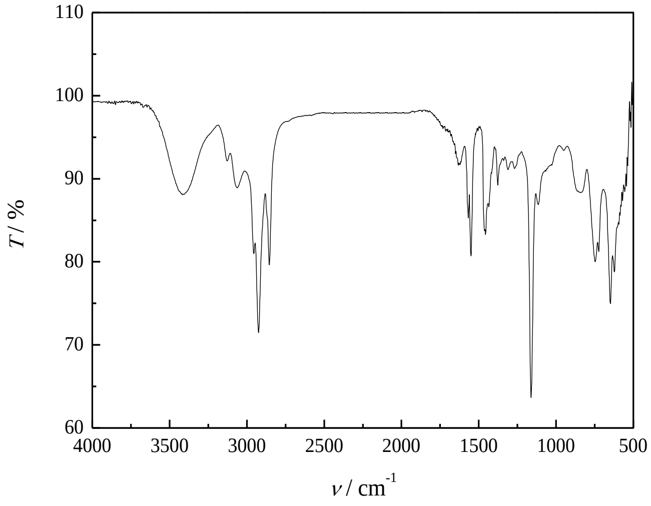

Figure S16. IR spectra of Poly(1-octyl-3-vinylimidazolium bromide) **3d**

IR neat: 3123 cm^{-1} (sym. stretching ν HC((4)C(5)H); 3059 cm^{-1} (sym. stretching *ν* of imidazole ring *ν* CH_3 HCH); 2956 cm^{-1}, 2924 cm^{-1} (asym. stretching *ν* C-H); 2854 (sym. stretching *ν* CH_3); 1625 cm^{-1} (stretching *ν* C=C); 1560 cm^{-1}, 1550 cm^{-1} (in-plane *ν* of imidazolium ring); 1469 cm^{-1}, 1372 cm^{-1} (C-N stretching of imidazole ring); 1156 cm^{-1} (sceletal *ν* of imidazolium ring); 741 cm^{-1} (out-of-plane C-H bending *ν* of imidazole ring); 649 cm^{-1} (C2-N-C5 bending *ν* of imidazole ring).

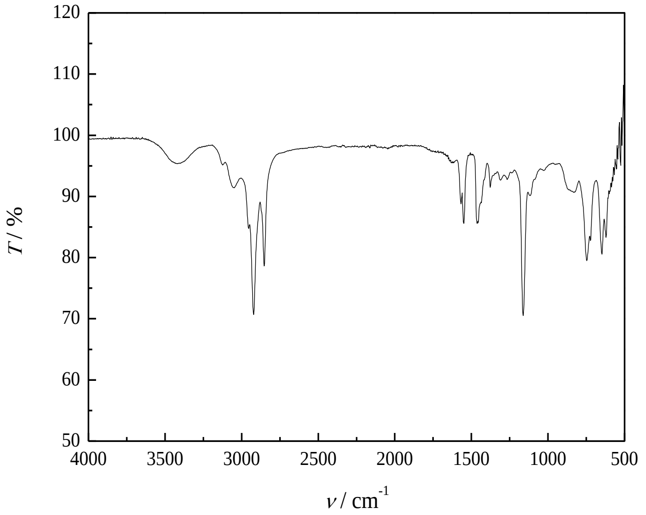

Figure S17. IR spectra of Poly(1-decyl-3-vinylimidazolium bromide) **3e**

IR neat: 3120 cm^{-1} (sym. stretching ν HC((4)C(5)H); 3056 cm^{-1} (sym. stretching *ν* of imidazole ring *ν* CH_3 HCH); 2927 cm^{-1} (asym. stretching *ν* C-H); 2850 cm^{-1} (sym. stretching *ν* CH_3); 1546 cm^{-1} (in-plane *ν* of imidazolium ring); 1463 cm^{-1} (C-N stretching of imidazole ring); 1159 cm^{-1} (sceletal *ν* of imidazolium ring); 746 cm^{-1} (out-of-plane C-H bending *ν* of imidazole ring); 650 cm^{-1} (C2-N-C5 bending *ν* of imidazole ring).

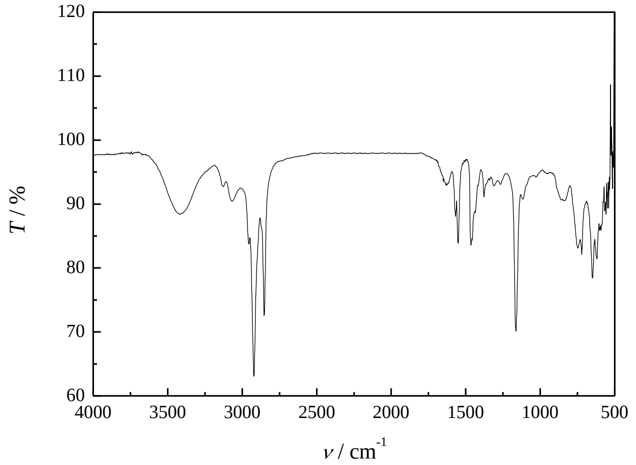

Figure S18. IR spectra of Poly(1-dodecyl-3-vinylimidazolium bromide) **3f**

IR neat: 3129 cm^{-1} (sym. stretching ν HC((4)C(5)H); 3059 cm^{-1} (sym. stretching *ν* of imidazole ring *ν* CH_3 HCH); 2917 cm^{-1} (asym. stretching *ν* C-H); 2851 cm^{-1}, 1374 cm^{-1} (sym. stretching *ν* CH_3); 1570 cm^{-1}, 1548 cm^{-1} (in-plane *ν* of imidazolium ring); 1466 cm^{-1} (C-N stretching of imidazole ring); 1161 cm^{-1} (sceletal *ν* of imidazolium ring); 741 cm^{-1} (out-of-plane C-H bending *ν* of imidazole ring); 649 cm^{-1} (C2-N-C5 bending *ν* of imidazole ring).

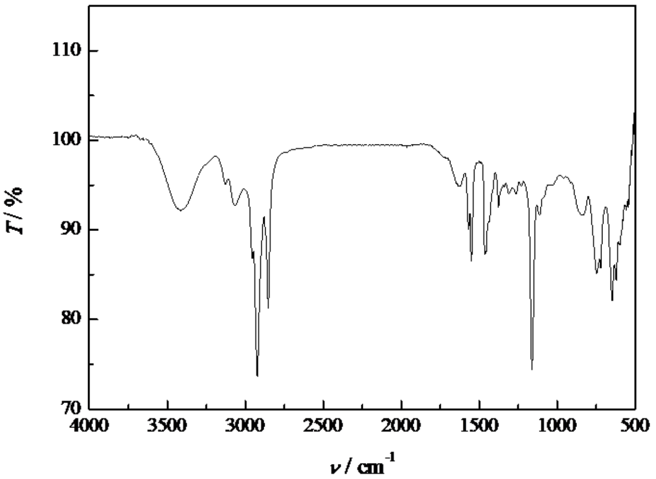

Figure S19. IR spectra of Poly(1-decyl-3-vinylimidazolium bromide) electrospun fibers

IR: 3128 cm^{-1} (sym. stretching ν HC((4)C(5)H); 3068 cm^{-1} (sym. stretching *ν* of imidazole ring *ν* CH_3 HCH); 2924 cm^{-1} (asym. stretching *ν* C-H); 2848 cm^{-1} (sym. stretching *ν* CH_3); 1554 cm^{-1} (in-plane *ν* of imidazolium ring); 1458 cm^{-1} (C-N stretching of imidazole ring); 1161 cm^{-1} (sceletal *ν* of imidazolium ring); 746 cm^{-1} (out-of-plane C-H bending *ν* of imidazole ring); 648 cm^{-1} (C2-N-C5 bending *ν* of imidazole ring).

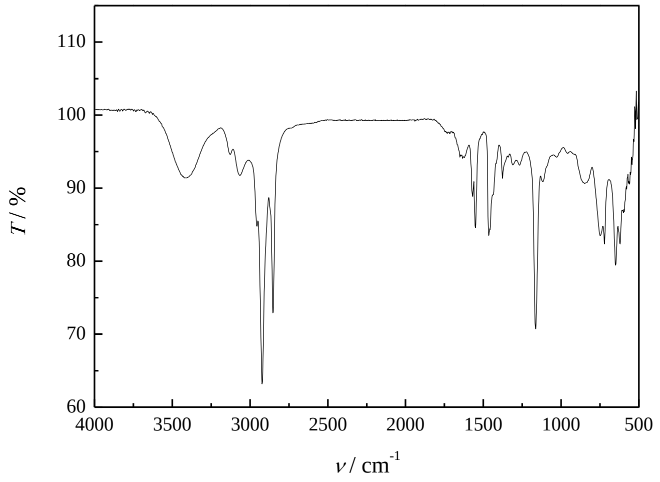

Figure S20. IR spectra of Poly(1-dodecyl-3-vinylimidazolium bromide) electrospun fibers

IR: 3128 cm^{-1} (sym. stretching ν HC((4)C(5)H); 3064 cm^{-1} (sym. stretching *ν* of imidazole ring *ν* CH_3 HCH); 2924 cm^{-1} (asym. stretching *ν* C-H); 2852 cm^{-1}, 1371 cm^{-1} (sym. stretching *ν* CH_3); 1570 cm^{-1}, 1548 cm^{-1} (in-plane *ν* of imidazolium ring); 1467 cm^{-1} (C-N stretching of imidazole

ring); 1161 cm^{-1} (sceletal ν of imidazolium ring); 752 cm^{-1} (out-of-plane C-H bending ν of imidazole ring); 653 cm^{-1} (C2-N-C5 bending ν of imidazole ring).

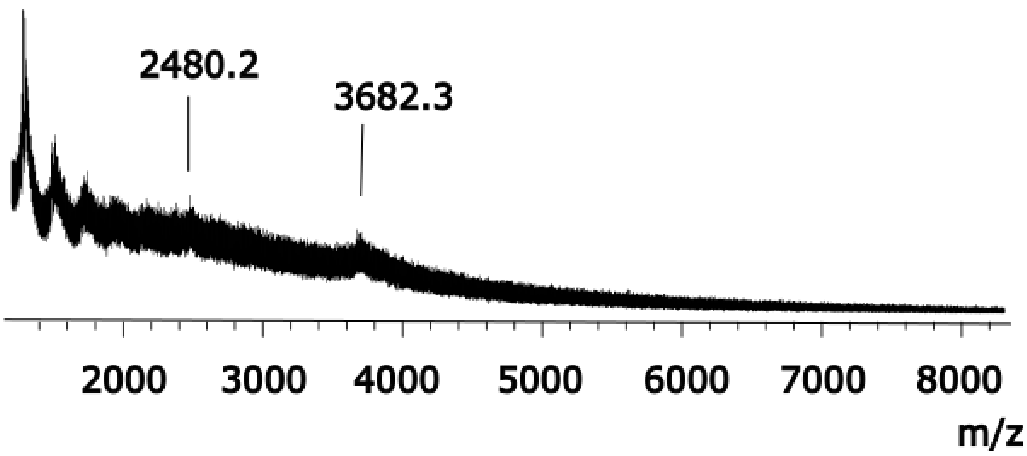

Figure S21. MS spectrum of Poly(1-ethyl-3-vinylimidazolium bromide) **3a**

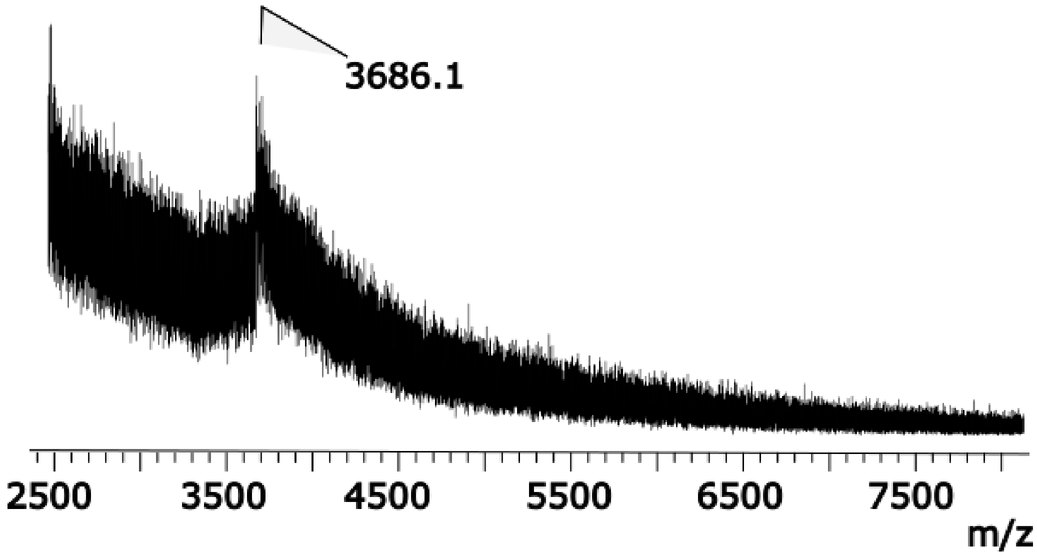

Figure S22. MS spectrum of Poly(1-butyl-3-vinylimidazolium bromide) **3b**

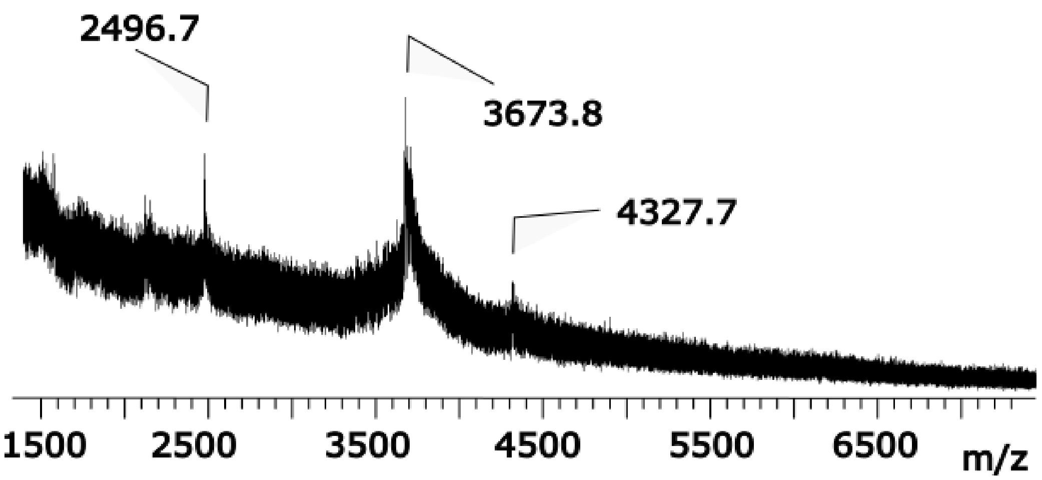

Figure S23. MS spectrum of Poly(1-hexyl-3-vinylimidazolium bromide) **3c**

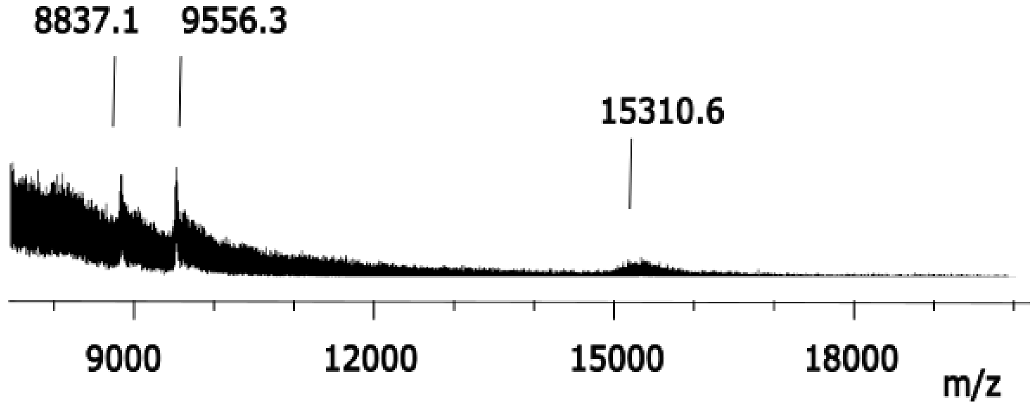

Figure S24. MS spectrum of Poly(1-octyl-3-vinylimidazolium bromide) **3d**

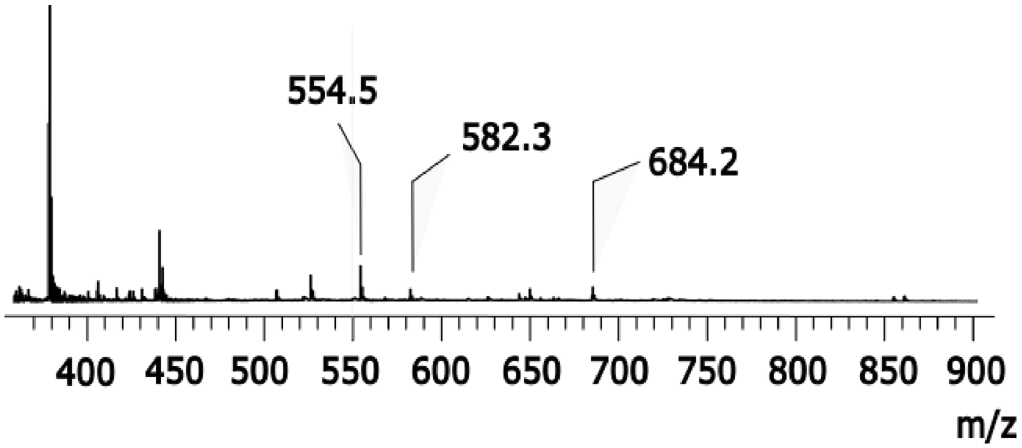

Figure S25. MS spectrum of Poly(1-decyl-3-vinylimidazolium bromide) **3e** (0.1% AIBN)

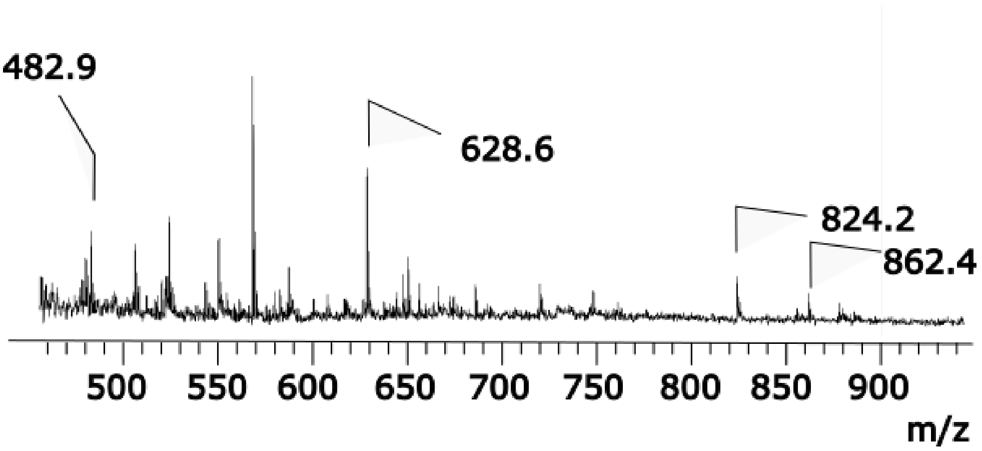

Figure S26. MS spectrum of Poly(1-decyl-3-vinylimidazolium bromide) **3e** (0.5% AIBN)

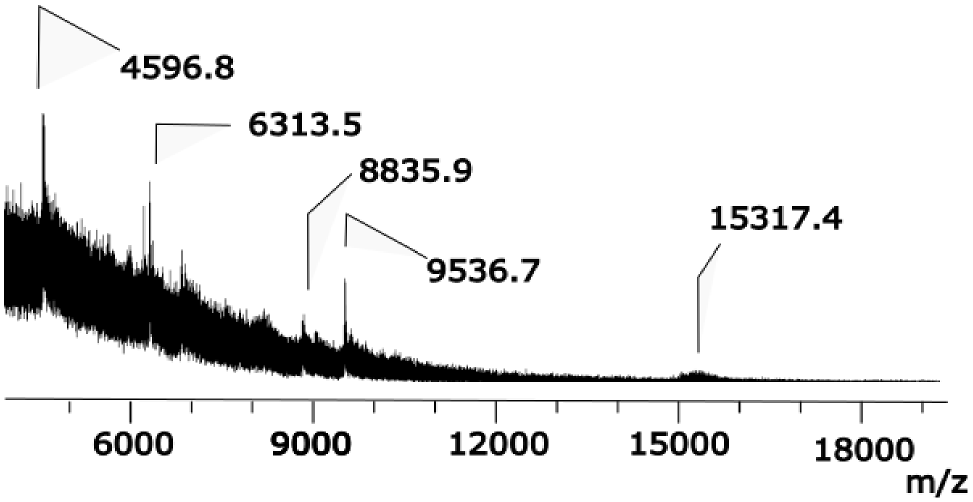

Figure S27. MS spectrum of Poly(1-decyl-3-vinylimidazolium bromide) **3e** (1% AIBN)

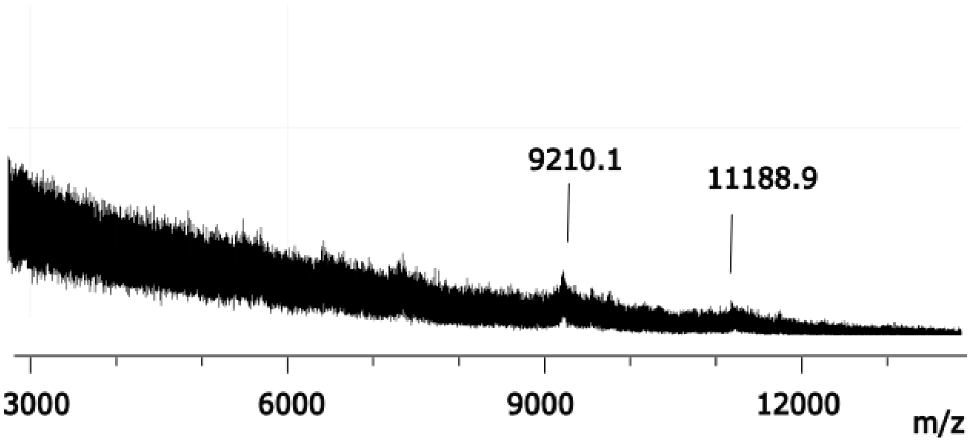

Figure S28. MS spectrum of Poly(1-dodecyl-3-vinylimidazolium bromide) **3f** 1% AIBN

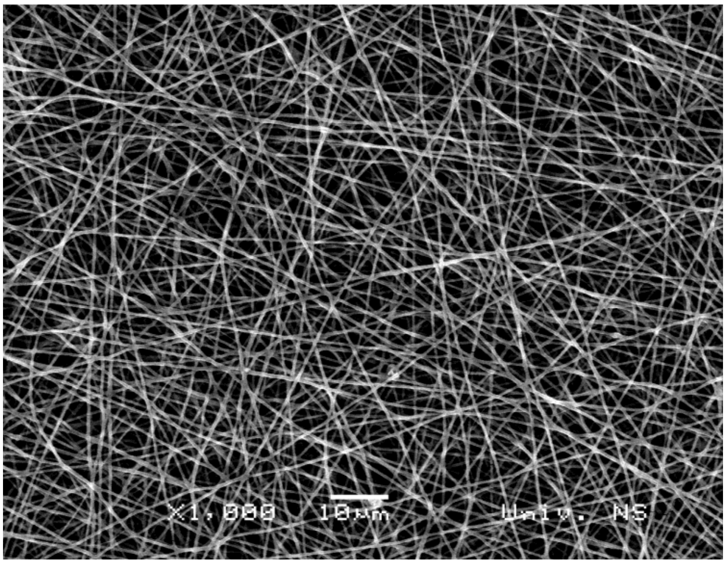

Figure S29. SEM micrograph of Poly([C$_{10}$VIm][Br]) fibers at magnification: X1000

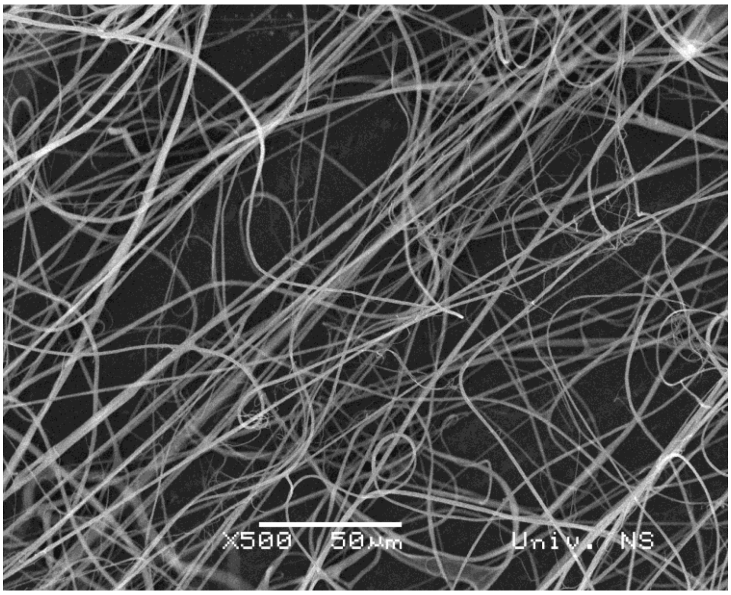

Figure S30. SEM micrograph of Poly([C$_{12}$VIm][Br]) fibers at magnification: X500
